# Observation of the 4π-periodic Josephson effect in indium arsenide nanowires

**DOI:** 10.1038/s41467-018-08161-2

**Published:** 2019-01-16

**Authors:** Dominique Laroche, Daniël Bouman, David J. van Woerkom, Alex Proutski, Chaitanya Murthy, Dmitry I. Pikulin, Chetan Nayak, Ruben J. J. van Gulik, Jesper Nygård, Peter Krogstrup, Leo P. Kouwenhoven, Attila Geresdi

**Affiliations:** 1 QuTech and Kavli Institute of Nanoscience, Delft University of Technology, 2600 GA Delft, The Netherlands; 2 Department of Physics, University of California, Santa Barbara, CA 93106 USA; 3 Station Q, Microsoft Research, Santa Barbara, CA 93106-6105 USA; 4 Center for Quantum Devices and Station Q Copenhagen, Niels Bohr Institute, University of Copenhagen, Universitetsparken 5, 2100 Copenhagen, Denmark; 5 Microsoft Station Q Delft, 2600 GA Delft, The Netherlands

## Abstract

Quantum computation by non-Abelian Majorana zero modes (MZMs) offers an approach to achieve fault tolerance by encoding quantum information in the non-local charge parity states of semiconductor nanowire networks in the topological superconductor regime. Thus far, experimental studies of MZMs chiefly relied on single electron tunneling measurements, which lead to the decoherence of the quantum information stored in the MZM. As a next step towards topological quantum computation, charge parity conserving experiments based on the Josephson effect are required, which can also help exclude suggested non-topological origins of the zero bias conductance anomaly. Here we report the direct measurement of the Josephson radiation frequency in indium arsenide nanowires with epitaxial aluminium shells. We observe the 4π-periodic Josephson effect above a magnetic field of ≈200 mT, consistent with the estimated and measured topological phase transition of similar devices.

## Abstract (web summary)

The interplay of induced superconductivity and magnetic fields should drive InAs nanowires into a topological superconducting phase. Laroche et al. use the microwave radiation emitted by an InAs nanowire Josephson junction to observe the 4π-periodic Josephson effect, a hallmark of the topological phase.

## Introduction

The universal relation between the frequency *f*_J_ of the oscillating current and an applied DC voltage bias *V* across a superconducting weak link^1^ is determined solely by natural constants.

1 $$\frac{{f_{\mathrm{J}}}}{V} = \frac{{2e}}{h} = {{\Phi }}_0^{ - 1} = 483.6\,{\mathrm{MHz}}\,{\mathrm{\mu V}}{\kern 1pt} ^{ - 1},$$

where *e* is the single electron charge, *h* is the Planck constant and *Φ*_0_ is the superconducting flux quantum. This relation, describing the conventional, 2π-periodic Josephson effect, can be understood as the tunneling of Cooper pairs with a net charge e^*^ = 2*e* coupled to photons of energy *hf*^2^. This coupling, referred to as the AC Josephson effect, has first been measured in superconducting tunnel junctions^3^ and has been shown to persist in metallic weak links^4^, carbon nanotubes^5^ and semiconductor channels^6,7^, as well as in high critical temperature superconductors^8^.

**Fig. 1:**
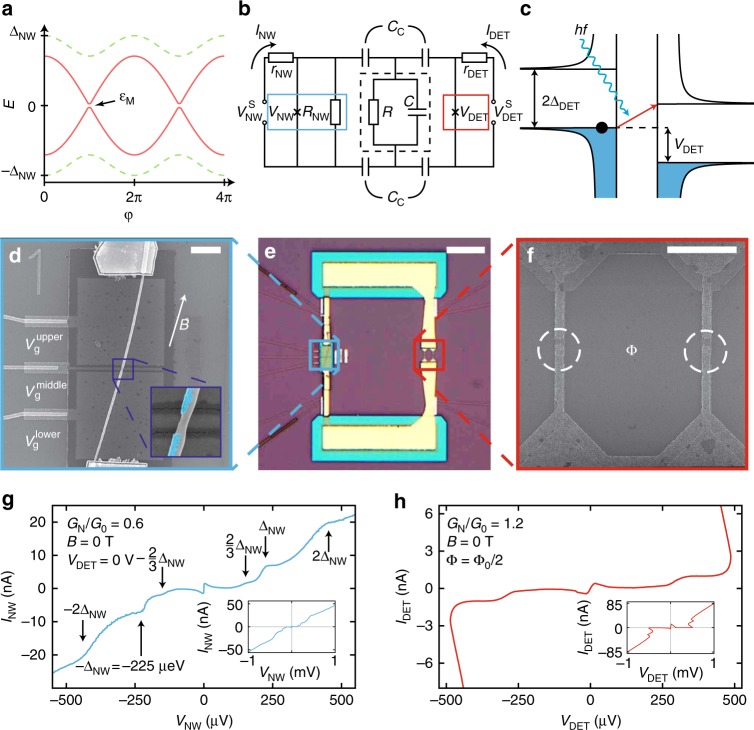
Principles of the experiment. **a** Energy dispersion of topologically trivial (dashed green line) and nontrivial (solid red line) Andreev levels inside a NW Josephson junction as a function of the phase difference across the junction. The gap *ε*_M_ arises from the finite MZM wavefunction overlap. **b** Equivalent circuit diagram of the device. The NW junction (in blue box) is capacitively coupled to the superconducting tunnel junction (red box) via the capacitors *C*_*C*_. The microwave losses and stray capacitance are modeled by the RC element enclosed by the dashed black box, see text. The applied DC bias voltages are $V_{{\mathrm{NW}}}^{\mathrm{S}}$ and $V_{{\mathrm{DET}}}^{\mathrm{S}}$ with an effective internal resistance *r*_NW_ and *r*_DET_, respectively. **c** Principle of the frequency-sensitive detection based on photon-assisted tunneling: an absorbed photon with an energy *hf* gives rise to quasiparticle current if *hf* > 2Δ_DET_ - *eV*_DET_. **d** Scanning electron micrograph of the NW junction placed on three electrostatic gates. A false color micrograph of the junction is shown in the inset, with the epitaxial Al shell highlighted in cyan. **e** Bright-field optical image of the coupling circuitry between the NW junction (blue box) and the detector junction (red box). **f** Micrograph of the split tunnel junction detector. The junctions are encircled. **g** Measured *I*_NW_(*V*_NW_) characteristics of the NW junction at zero in-plane magnetic field exhibiting a supercurrent branch and multiple Andreev reflections. **h** Measured *I*_DET_(*V*_DET_) trace of the detector split junction at zero in-plane magnetic field with a minimized switching current. The insets in (**g**) and (**h**) show the large scale *I*(*V*) trace of each junction. The normal state conductance *G*_N_ is given in the units of *G*_0_ = 2*e*^2^/*h*. All images and data were taken on device NW1. The scale bars denote 1 μm (**d**), 10 μm (**e**), and 1 μm (**f**), respectively

In proximitized semiconductor nanowires, an effective superconducting gap with a p-wave symmetry arises due to the breaking of the time-reversal symmetry above a threshold magnetic field^9–16^. When a weak link is formed between two leads, the p-wave component leads to a factor of two increase in the flux periodicity, giving rise to the so-called 4π-periodic Josephson effect^17,18^. Phenomenologically, this phase periodicity is equivalent to an effective tunneling charge e^*^ = *e* instead of 2*e* in Eq. (1). Therefore, in this Majorana zero mode (MZM) regime, the frequency at a given voltage bias *V* drops by a factor of two, *f*_MZM_(*V*) = *f*_J_(*V*)/2, providing a robust signature of the topological phase transition in the superconducting leads. In real devices, however, the finite size of the topological regions^19^, poisoning events^9,18^ and Landau–Zener (LZ) tunneling to the quasiparticle continuum^20^ can effectively restore the 2π-periodic trivial state. The latter two parity-mixing effects cause the system to relax to its ground state, effectively constraining the system in the lowest topological energy branch (red solid lines in Fig. 1a). Nevertheless, out-of-equilibrium measurements performed at rates faster than these equilibration processes can still capture the 4π-periodic nature of topological junctions^19–21^. In contrast, finite-size effects can be avoided by biasing the junction at voltages large enough to overcome the Majorana hybridization gap *ε*_M_^20^.

Here, we report the direct observation of a magnetic field-induced halving of the Josephson radiation frequency^22^ in indium arsenide nanowire (InAs NW) junctions partially covered with an epitaxially grown aluminium shell (Fig. 1d). In this system, possessing a hard induced superconducting gap^23^, previous direct transport experiments suggest parity lifetimes above 0.1 μs^24^ and hybridization energies *ε*_M_ ≲ 1 μeV for leads longer than 1.5 μm^25^. Thus, a frequency-sensitive measurement in the microwave domain is expected to reveal the 4π-periodic Josephson effect^26,27^.

## Results

### Frequency-sensitive detection of Josephson radiation

As a frequency-sensitive microwave detector, we utilized a superconducting tunnel junction with a quasiparticle gap of Δ_DET_, wherein the photon-assisted electron tunneling (PAT) current contributed to the DC current above a voltage bias threshold *eV*_DET_ > 2Δ_DET_ - *hf*^22,28^ (Fig. 1c). This on-chip detector^29^, coupled via capacitors *C*_*C*_ to the NW junction (see Fig. 1b for the schematics and Fig. 1e for an optical image of the device), was engineered to result in an overdamped microwave environment characterized by a single *f*_c_ = (2π*RC*)^-1^ ≈ 28 GHz cutoff frequency with *R* = 538 Ω and *C* = 10.4 fF (see Supplementary Fig. 2). The resulting broadband coupling to the detector^7^ inhibited higher order photon emission, which could mimic the 4π-periodic Josephson effect^30^.

The nanowire was deterministically deposited on a set of three gates covered by 30-nm thick SiN_*x*_ dielectric as shown in Fig. 1d. The Josephson weak link, where the Al shell was removed by wet chemical etching, was located above the central gate (see inset of Fig. 1d). We investigated devices with junction lengths ranging from 86 to 271 nm. The high quality of the nanowire junction is apparent from the presence of distinct multiple Andreev reflection steps in its *I*_NW_(*V*_NW_) characteristics^31^ (Fig. 1g), which is a signature of the hard superconducting gap in the nanowire^23^. The observed curves and linear conductance also establish that no conductive mode with a transmission close to unity exists in the channel, which could contribute to the 4π-periodic signal even in the absence of topological ordering^20^.

The microwave detector, presented in Fig. 1f, was fabricated using two angle-evaporated^32^ Al/AlO_*x*_/Al tunnel junctions, forming a superconducting quantum interference device. This geometry allowed us to minimize the Josephson energy of the detector by applying *Φ* = *Φ*_0_/2 flux through the loop (see Fig. 1h) and thus to limit its back action to the nanowire. The respectively 8- and 11-nm thick Al layers set an in-plane critical magnetic field of the detector in excess of 1 T, well above the measured topological transition in similar devices^25^. Nevertheless, increasing subgap currents limited our experimental field range to 325–650 mT for different devices. The circuit parameters and fabrication details are given in the Supplementary Tables and in the Methods section, respectively.

In the presence of a voltage spectral density *S*_*V*_(*f*), the DC current contribution of the PAT process is as follows^22,28^ in the subgap regime, where *eV*_DET_ < 2Δ_DET_:

2 $$I_{{\mathrm{PAT}}}\left( {V_{{\mathrm{DET}}}} \right) = {\int}_0^\infty {\kern 1pt} {\mathrm{d}}f\left( {\frac{e}{{hf}}} \right)^2S_V(f)I_{{\mathrm{QP,0}}}\left( {V_{{\mathrm{DET}}} + \frac{{hf}}{e}} \right).$$

Here, *I*_*QP*,0_(*V*_DET_) is the tunnel junction current in the absence of absorbed radiation, *S*_*V*_(*f*) = 0 (see Fig. 1h). Note that the quasiparticle gap edge at *eV*_DET_ = 2Δ_DET_ resulted in a sharp increase of *I*_*QP*,0_(*V*_DET_). In the presence of monochromatic radiation with a frequency *f*_0_, *S*_*V*_(*f*) ~ *δ*(*f* - *f*_0_), *I*_PAT_(*V*_DET_) thus developed a step-like feature at *hf*_0_ = 2Δ_DET_ - *eV*_DET_. With a phenomenological effective charge e^*^ of the AC Josephson effect, we write this condition in terms of the voltage drop on the nanowire, *V*_NW_.

3 $$e^ \ast V_{{\mathrm{NW}}} = hf_0 = 2{\mathrm{\Delta }}_{{\mathrm{DET}}} - eV_{{\mathrm{DET}}},$$

where e^*^ = 2*e* for conventional junctions (see Eq. (1)) and e^*^ = *e* in the 4π-periodic regime. To extract e^*^ and thus determine the periodicity of Josephson radiation, we tracked the transconductance peak d*I*_PAT_/d*V*_NW_(*V*_NW_, *V*_DET_) measured by standard lock-in techniques at a frequency of 17.7 Hz (see Supplementary Fig. 1). The experiments were performed at the base temperature of a dilution refrigerator (~20 mK).

### Josephson radiation as a function of the magnetic field

**Fig. 2:**
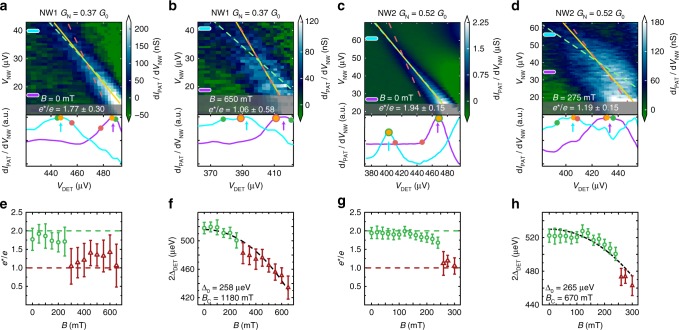
Magnetic field-induced 4π-periodic Josephson radiation. Differential transconductance d*I*_PAT_/d*V*_NW_ as a function of *V*_NW_ and *V*_DET_ for devices NW1 (**a**, **b**) and NW2 (**c**, **d**) at zero and finite magnetic fields, respectively. The position of the transconductance peak maps the frequency of the monochromatic Josephson radiation. A linear fit e^*^V_NW_ = 2Δ_DET_ - *eV*_DET_ through these peaks is shown as an orange line. Dashed green and red lines show linear fits with a fixed slope corresponding to e^*^ = 2*e* and e^*^ = *e*, respectively. The shaded regions show the regimes where the fit of the transconductance peak is not reliable, see text. Two normalized and smoothed horizontal linecuts are plotted, where arrows point to the position of the extracted peaks. The orange, green, and red dots denote the position of the best fit, the e^*^ = 2*e* fit, and the e^*^ = *e* fit, respectively. The evolution of e^*^(*B*) and 2Δ_DET_(*B*) is presented in (**e**, **f**) for NW1 and in (**g**, **h**) for NW2. For the calculation of the error bars, see text and Supplementary Note 2. The transition from the 2π- to 4π-periodic Josephson radiation is observed between 175 and 300 mT as e^*^ evolves from values near 2*e* (green circles) to values close to 1*e* (red triangles). For all devices, 2Δ_DET_(*B*) drops monotonically (black dashed line, see text), independently of the change in e^*^

**Fig. 3:**
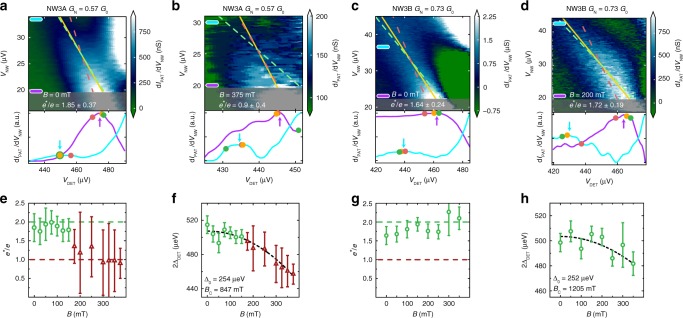
Gate tuning of the 4π-periodic radiation regime. Differential transconductance d*I*_PAT_/d*V*_NW_ as a function of *V*_NW_ and *V*_DET_ for device NW3 at gate setting A (**a**, **b**) and setting B (**c**, **d**) at zero and finite magnetic fields, respectively. A linear fit and fits with fixed slopes e^*^ = 2*e* and e^*^ = *e* are shown as an orange line, a dashed green line, and a dashed red line, respectively. Two normalized and smoothed horizontal linecuts are also presented, in which the arrows point to the position of the extracted peaks. The evolution of e^*^(*B*) and Δ_DET_(*B*) is shown in (**e**, **f**) for setting A and in (**g**, **h**) for setting B. A transition from 2π- to 4π-periodic Josephson radiation is observed for gate setting A, but the radiation remains 2π-periodic for setting B. The gate voltage values are shown in Supplementary Table 2. For the calculation of the error bars, see text and Supplementary Note 2

Typical experimental datasets are shown in Fig. 2 for two nanowire junctions (NW1 and NW2, respectively) as the source of Josephson radiation. We limited the detector voltage range by the condition d*I*_DET_/d*V*_DET_ < 10 μS, where the subgap quasiparticle current was still negligible, typically *I*_DET_ ≲ 1 nA. A lower limit of the emitter junction voltage was defined by the phase diffusion regime^33^ characterized by periodic switching and retrapping events, which breaks the validity of Eq. (1) (see Supplementary Note 3). We therefore did not consider the low *V*_NW_ regime, within the supercurrent peak. We show this range, excluded from the linear fits, shaded in gray in Figs. 2 and 3 (see Supplementary Note 2 on the characterization of these limits). We fit the peak positions using Eq. (3) in order to extract e^*^ and Δ_DET_ as a function of the applied in-plane magnetic field. The typical standard deviation was 3.6 GHz for each frequency data point (see Supplementary Note 2). The error bars of the fitted parameters were determined using the bootstrapping method^34^ (see Supplementary Note 2), and they show the full width at half maximum yielding a confidence level of 75% for a Gaussian lineshape.

At zero magnetic field (Fig. 2a, c), the emitted Josephson radiation is always 2π-periodic with an extracted effective charge close to e^*^ = 2*e*, as shown by the good agreement between the orange line and the dashed green line (best fit with fixed e^*^ = 2*e*). In contrast, NW1 and NW2 exhibit the 4π-periodic Josephson effect above a threshold magnetic field (Fig. 2b, d), where e^*^ ≈ *e*. The full evolution is shown in Fig. 2e, g, where a sharp transition is visible from e^*^ ≈ 2*e* (green circles) to e^*^ ≈ *e* (red triangles). Finally, the fitted Δ_DET_ (Fig. 2c, f) shows a monotonic decrease described by $2{\mathrm{\Delta }}_{{\mathrm{DET}}}(B) = 2{\mathrm{\Delta }}_0\sqrt {1 - B^2{\mathrm{/}}B_{\mathrm{c}}^2}$ for all devices (dashed lines), with no additional feature at the transition field. In contrast with the nanowire junctions, our control device, an Al/AlO_*x*_/Al tunnel junction, exhibits no transition in e^*^ over the entire magnetic field range (see Supplementary Fig. 5).

### Josephson radiation at different gate voltages

Figure 3 shows the magnetic field evolution of device NW3 at two distinct gate settings with similar *G*_N_ and d*I*_PAT_/d*V*_NW_ corresponding to similar Josephson couplings. By tuning the chemical potential in the nanowire via changing the gate voltages, it is possible to displace the position of the onset of the 4π-periodic Josephson radiation from ≈175 mT (Fig. 3b) to values larger than 375 mT (Fig. 3d). Note that the additional local maximum at high *V*_NW_ values, also observed in earlier experiments^7^, is attributed to the shot noise of the nanowire junction.

The possibility to tune the nanowire devices into the 4π-periodic Josephson radiation regime with both magnetic field and chemical potential is consistent with the predicted phase diagram of this system^9,10,18^. We observed the same behavior in four distinct nanowire devices (see Supplementary Fig. 4 for device NW4), which we could interpret within the single sub-band model of the topological phase transition that takes place at a magnetic field B^*^, where $E_{\mathrm{z}} = g\mu _{\mathrm{B}}B^ \ast {\mathrm{/}}2 = \sqrt {{\mathrm{\Delta }}_{{\mathrm{NW}}}^2 + \mu _{{\mathrm{NW}}}^2}$. Here *g* and *μ*_B_ are the Landé g factor and the Bohr magnetron, respectively. From our device parameters (see Supplementary Table 2), lower bounds on the g factors ranging from *g* ≈ 11 (B^*^ = 175 mT) in device NW3 to *g* ≈ 35 (B^*^ = 190 mT) in device NW4 were obtained, in agreement with the values reported in similar devices^15,25,35^. In contrast, an accidental crossing of a trivial Andreev bound state would be inconsistent with the observed field range of Δ*B* ~ 0.3 T of the 4π-periodic radiation, since within this range, a spinful Andreev level^35^ would evolve over the scale of the superconducting gap, Δ_NW_ ~ *gμ*_B_Δ*B*, suppressing the 4π periodicity. We, however, did not observe a continuous variation of the onset magnetic field B^*^ as a function of the applied gate voltages. This behavior is consistent with the calculations of the topological phase diagram based on realistic device simulations including orbital effects of the magnetic field^36^ and multiple spatial dimensions^37,38^ of the device.

We observed a single Josephson radiation frequency in the 4π-periodic regime, which was consistent with the supercurrent being predominantly carried by a single transmitting mode. While we were not able to reliably extract the transparency and the number of modes in our devices, the single-mode regime was observed earlier in similar InAs nanowires^35,39,40^. We also note that an upper bound on the channel transmission of *τ* = *G*_N_/*G*_0_ could be determined from the normal state conductance *G*_N_ < *G*_0_, which was measured in the linear regime well above the superconducting gap. This value is shown in Figs. 2 and 3 for each device.

### Numerical simulations of the Josephson radiation frequency

Next, we numerically evaluated the expected voltage spectral density seen by the detector junction in various regimes. We used the quasiclassical resistively and capacitively shunted junction model coupled to a stochastic differential equation describing the occupation of the single pair of Andreev levels in the NW junction. The equivalent circuit of the device in the microwave domain is shown in Fig. 1b, in which each element is experimentally characterized^7^ (see Supplementary Fig. 2 and Supplementary Tables). Note that we neglected the load of the detector on the circuit, which is justified by its negligible subgap conductance compared to that of all other elements in the circuit.

Our model of the nanowire junction considers LZ tunneling between branches of the energy-phase dispersion shown in Fig. 1a, as well as tunneling to the continuum and stochastic quasiparticle poisoning events^20^. The probability of LZ tunneling is determined by the voltage drop *V*_NW_ according to *P*_LZ_ = exp(-*V*_0_/*V*_NW_), where $eV_0 = 4\uppi\varepsilon _{\mathrm{M}}^2{\mathrm{/}}\left( {{\mathrm{\Delta }}_{{\mathrm{NW}}}\sqrt \tau } \right)$ is the characteristic voltage above which *P*_LZ_ ~ 1. In this limit, 4π periodicity is observed despite the gap *ε*_M_ caused by finite-size effects^25^. Similarly, LZ tunneling to the continuum close to *φ* = 2π defines a voltage scale $eV_1 = 2{\mathrm{\pi\Delta }}_{{\mathrm{NW}}}\left( {1 - \sqrt \tau } \right)^2{\mathrm{/}}\sqrt \tau$, above which 2π periodicity is restored^20^. We note that a trivial Andreev bound state in the short junction limit can be modeled similarly with *eV*_0_ =π Δ_NW_(1 - *τ*) and *eV*_1_ = 0.

**Fig. 4:**
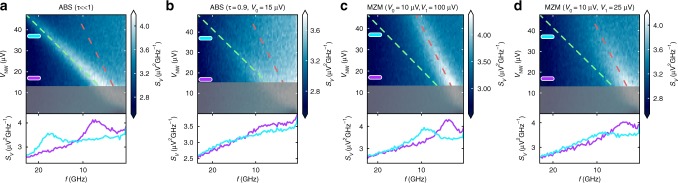
The calculated radiation spectrum. The voltage spectral density *S*_*V*_(*f*) incident on the detector junction, computed by numerically solving the system of stochastic differential equations shown in Supplementary Note 3. **a**, **b** Show results for a junction in the trivial regime (small transmission and large transmission, respectively), while (**c**, **d**) show the emission spectrum in the topological regime. *V*_0_ and *V*_1_ are voltage scales for Landau–Zener tunneling between branches of the junction bound state and for tunneling to the quasiparticle continuum, respectively; see text. Circuit parameters are set as *r*_NW_ = 2.4 kΩ, *R*_NW_ = 50 kΩ, *R* = 0.5 kΩ, *C* = 10 fF, *C*_*C*_ = 400 fF, and $I_{{\mathrm{NW}}}^{\mathrm{0}} = 8{\kern 1pt}$ nA. The noise temperature *T* = 150 mK and the quasiparticle poisoning rate is Γ_*q*_ = 100 MHz. As in Fig. 2, the dashed green and red lines show the frequency of Josephson radiation corresponding to e^*^ = 2*e* and e^*^ = *e*, respectively. The estimated phase-diffusion region is shaded in gray

Figure 4 shows representative plots obtained by numerically evaluating *S*_*V*_(*f*, *V*_NW_) (see Supplementary Note 3), which determines the photon-assisted tunneling current by Eq. (2). We observed that the numerical results agreed well with the characteristic features of the experimental data. We found that the circuit equations allowed for a phase-diffusion regime at low *V*_NW_ values^33^, where e^*^V_NW_ < *hf*, because the junction spent part of the time in the steady supercurrent state in which the voltage drop was zero. The calculations also reproduced the absence of higher harmonics in the radiation spectrum, attributed to the low transmission of the junction and the overdamped nature of the microwave environment^30^. This confirms our expectation of the suppression of multiphoton processes due to a low-quality factor, justifying the usage of the semiclassical junction model.

A key result of these simulations in a wide range of junction parameters is that with the circuit elements taking values representative of those in the experiment, the radiation frequency always reflects the internal dynamics of the nanowire Josephson junction both in the 2π-periodic (Fig. 4a, b) and in the 4π-periodic emission regimes (Fig. 4c, d). Finally, we note that our results are consistent with *V*_0_ ≲ 15 μeV translating to an avoided crossing *ε*_M_ ≲ 10 μeV. Using the exponential cutoff in ref. ^25^, this suggests that our devices have a continuous topological region of several hundreds of nanometers on each side of the nanowire junction, which is consistent with the scanning electron microscopic images of the devices.

## Discussion

In conclusion, we observed the 4π-periodic Josephson effect in multiple InAs nanowires above a threshold magnetic field in a range of 175–300 mT. This effect, which can be suppressed by tuning the gate voltages, is consistent with the expected signatures of a topological phase transition. By observing the periodicity of Josephson effect using an on-chip microwave detector, we investigated this system while preserving its charge parity, in line with the requirements for prospective topological quantum computers. This experimental technique may also prove instrumental in identifying more exotic non-Abelian anyon states^41,42^, due to its proven sensitivity to the periodicity of Josephson effect, directly measuring the charge fractionalization of the anyon state^43,44^.

## Methods

### Device fabrication

The devices were fabricated on commercially available undoped Si substrates with a 285-nm thick insulating SiO_*x*_ layer in a similar fashion as in refs. ^7,35^. All etching and metal-deposition steps were realized using standard positive-tone electron-beam lithography techniques. At first, three Ti/Au (5 nm/15 nm) electrostatic gates and the coupling capacitor bottom plates were deposited (see Fig. 1 for design details). These were subsequently covered by an ~30-nm thick SiN_*x*_ dielectric layer deposited by sputtering. Eleven 100-nm wide Cr/Pt (5 nm/25 nm) tracks were then defined. These ~100 Ω μm^*-*1^ resistive lines connected the gates, the (yet to be defined) Al/AlO_*x*_/Al detector, and the nanowire to the instrumentation setup. Next, the Al/AlO_*x*_/Al Josephson junctions were fabricated by evaporating 8- and 11-nm thick Al layers with an intermediate in-situ oxidation step at 0.5 mbar for 4 min using the Dolan bridge technique^32^. The nanowires were then deterministically deposited onto the electrostatic gates with a micro-manipulator setup equipped with an optical microscope. A gap in the nanowire Al shell was then created by wet etching using Transene D at a temperature of 48.2 °C for 12 s. Next, both the nanowire and the detector junctions were connected to the resistive lines with an 80-nm thick sputtered NbTiN film after an in-situ Ar plasma milling step. Finally, a Ti/Au (15/100 nm) layer was evaporated to define quasiparticle traps, the upper capacitor plates, and the contact pads. We note that no NbTiN film was used in device NW3. Instead, a Ti/Au (15/100 nm) layer was used to contact the nanowire and the detector. The dimensions and properties of each device are presented in Supplementary Table 1, and the experimental setup is described in Supplementary Fig. 1. We note that the detector was made of narrow and thin aluminum sections (see Fig. 1f) to limit the presence of vortices near the Al/AlO_*x*_/Al junctions, and thus to decrease the subgap current in finite magnetic field.

The InAs nanowires used in this work were grown via a two-step process by molecular beam epitaxy. The InAs nanowires were grown at 420 °C using the vapor–liquid–solid method with Au droplets as the catalyst. After cooling the system to *-*30 °C, Al was epitaxially grown on two of the six nanowire facets^23^.

### The microwave environment of the InAs Josephson junction

We modeled the effective microwave environment of the nanowire Josephson junction with a parallel lumped resistor (R) and capacitor (C) element, which accounted for the low-pass nature of the coupling circuit (see inset of Supplementary Fig. 2a). We determined the effective RC values by measuring a sample in which the nanowire junction was replaced by an Al/AlO_*x*_/Al tunnel junction. The supercurrent peak was fitted against the Ivanchenko–Zil’berman model to find the RC values and the noise temperature of the circuit^33^ at zero magnetic field (see Supplementary Fig. 2a). The critical current as a function of the magnetic field was then found using the same model, with the RC and the noise temperature fixed at their zero field values (Supplementary Fig. 2b). We note that the same coupling circuit was used in ref. ^7^, leading to RC and noise temperature values in good agreement with the current ones. Thus, we conclude that the reproducibility is good for all the samples featured in the current study. These parameters are used to theoretically study the dynamics of Josephson radiation.

### Reproducibility of the transition for nanowire devices

Supplementary Figure 3 shows every differential transconductance color plot from which the effective charge e^*^ has been extracted in Fig. 2e. The color plots nominally follow the same trend as the ones presented in Fig. 2. Supplementary Figure 4 shows the magnetic field evolution of e^*^ in device NW4. Device NW4 also exhibits a transition from 2π- to 4π-periodic Josephson radiation at *B* ~ 175 mT. As such, the observation of a magnetic field-induced transition in the periodicity of Josephson radiation has been observed in four distinct devices, showcasing the reproducibility of the observation.

### Josephson radiation of an Al/AlO_*x*_/Al tunnel junction

Supplementary Figure 5 shows our measured data with a conventional Al/AlO_*x*_/Al superconducting tunnel junction as the source of Josephson radiation. Evaluating e^*^ as a function of magnetic field in the same range as for Fig. 2 and Supplementary Fig. 4, we observed no transition in the periodicity of Josephson effect, confirming that the 4π-periodic Josephson radiation occurs only in nanowire junctions. We note that, in order to keep the circuit behavior similar, the normal state conductance of the tunnel junction was set to *G*_N,T_ = 0.26*G*_0_.

## Supplementary information

Supplementary Information

## Data Availability

The datasets analyzed during this study are available at the 4TU.ResearchData repository 10.4121/uuid:1f936840-5bc2-40ca-8c32-1797c12cacb1 (ref. ^45^).
