# Supplementary material for: Observation of the 4π-periodic Josephson effect in indium arsenide nanowires
Source: Nat Commun. 2019 Jan 16;10:245. doi: 10.1038/s41467-018-08161-2 (PMC6335404; doi:10.1038/s41467-018-08161-2)
Supplement: Supplementary file 1 — Supplementary Information [file 41467_2018_8161_MOESM1_ESM.pdf]

Supplementary Information
Observation of the 4π-periodic Josephson effect in indium arsenide nanowires

Dominique Laroche,[1,*] Daniël Bouman,[1,*] David J. van Woerkom,[1] Alex Proutski,[1] Chaitanya Murthy,[2] Dmitry I. Pikulin,[3] Chetan Nayak,[2,3] Ruben J. J. van Gulik,[1] Jesper Nygård,[4] Peter Krogstrup,[4] Leo P. Kouwenhoven,[1,5] and Attila Geresdi[1,†]

[1]*QuTech and Kavli Institute of Nanoscience, Delft University of Technology, 2600 GA Delft, The Netherlands*

[2]*Department of Physics, University of California, Santa Barbara, CA 93106, USA*

[3]*Station Q, Microsoft Research, Santa Barbara, California 93106-6105, USA*

[4]*Center for Quantum Devices and Station Q Copenhagen, Niels Bohr Institute, University of Copenhagen, Universitetsparken 5, 2100 Copenhagen, Denmark*

[5]*Microsoft Station Q Delft, 2600 GA Delft, The Netherlands*

[*] These authors contributed equally to this work.

[†] To whom correspondence should be addressed; E-mail: a.geresdi@tudelft.nl

CONTENTS

Supplementary Figures 3

Supplementary Tables 8

Supplementary Note 1: Determination of the superconducting gaps 10

Supplementary Note 2: Peak finding and fitting 12

Supplementary Note 3: Theory 15
- Probabilistic model of voltage biased Majorana Josephson junction 15
- Power spectrum for fixed junction voltage (no external circuit) 16
- Modeling the junction environment: equivalent circuit 19
- Circuit equations in the topological regime 25
- Numerical solution of circuit equations 27
- Derivation of power spectrum for fixed junction voltage 30
- Derivation of circuit equations 32

Supplementary References 33

SUPPLEMENTARY FIGURES

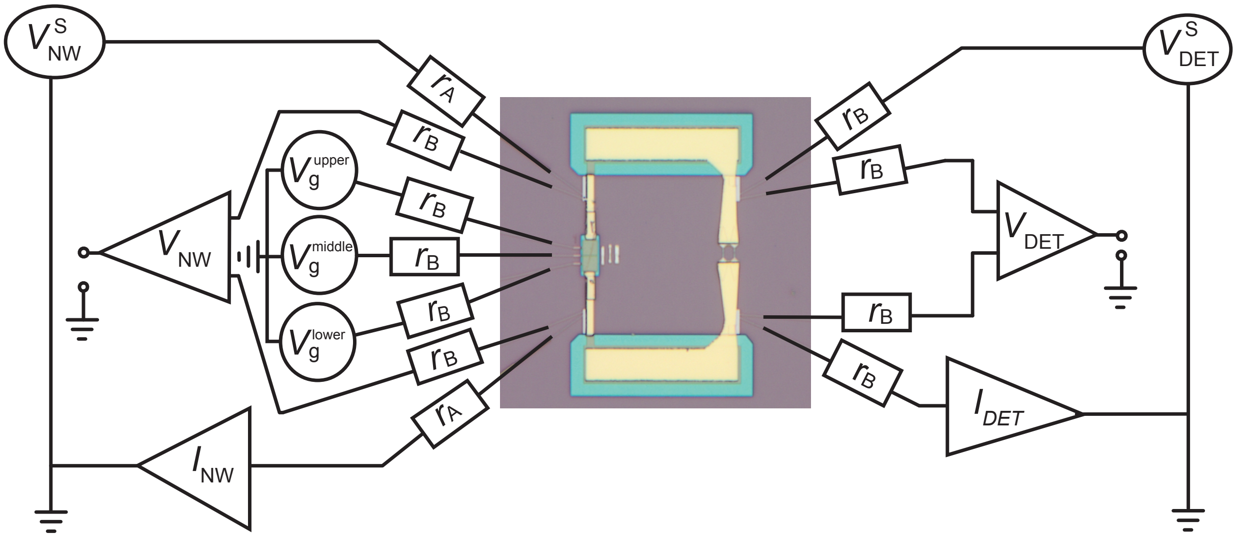

Supplementary Figure 1: **Schematics of the PAT measurement.** The voltage V_{NW}^{S} is the sum of a DC voltage and a 2 µV AC component at 17.7 Hz applied via resistive lines with $r_A = 1.2\,\mathrm{k\Omega}$ resistances. The voltage drop over the nanowire V_{NW} is measured in a four point probe geometry via the $r_B = 12\,\mathrm{k\Omega}$ on-chip resistors using a home-built low noise preamplifier. The current I_{NW} is measured simultaneously using a home-built current to voltage converter. The gate voltages V_g^{upper}, V_g^{middle} and V_g^{lower} are all applied via $r_B = 12\,\mathrm{k\Omega}$ on-chip resistors. The detector junction is measured in a similar, four point probe geometry, using $r_B = 12\,\mathrm{k\Omega}$ in all connecting lines. The DC current, originating from both the intrinsic characteristics of the detector and from PAT, and the AC component, generated solely by PAT, are measured with a home-built current to voltage converter. We note that it was not possible to perform the measurements in the 4-point geometry on the detector side of device NW4, nor on either side of device NW3. In these cases, series resistances of 116, 7.5 and 7.4 kΩ were taken into account to calculate V_{DET} from $V_{\mathrm{DET}}^{\mathrm{S}}$ in device NW4 and NW3, and to calculate V_{NW} from $V_{\mathrm{NW}}^{\mathrm{S}}$ in device NW3, respectively.

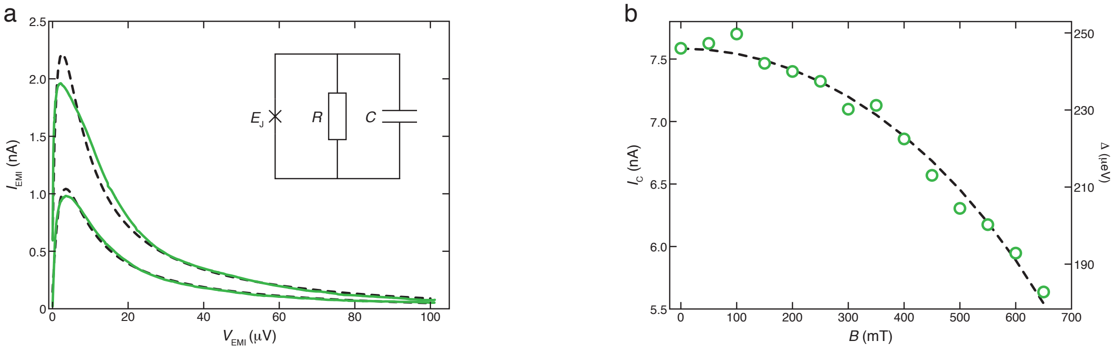

Supplementary Figure 2: **Characterization of the microwave environment.** **(a)** Supercurrent peak of an Al/AlO$_x$/Al junction emitter device at magnetic fields $B = 0\,\mathrm{mT}$ and $B = 650\,\mathrm{mT}$ (higher and lower solid curves respectively), with the dashed lines being the best fit yielding $R = 538\,\Omega$, $C = 10.4\,\mathrm{fF}$ and $T = 133\,\mathrm{mK}$. **(b)** Extracted critical current I_C as a function of B. The right axis shows the extracted superconducting gap $\Delta = 2I_\mathrm{C}/\pi G_\mathrm{N}$. The dashed line shows the best fit to $\Delta(B)/\Delta(B=0) = \sqrt{1 - B^2/B_c^2}$, yielding a critical field $B_{\mathrm{c,EMI}} = 953\,\mathrm{mT}$.

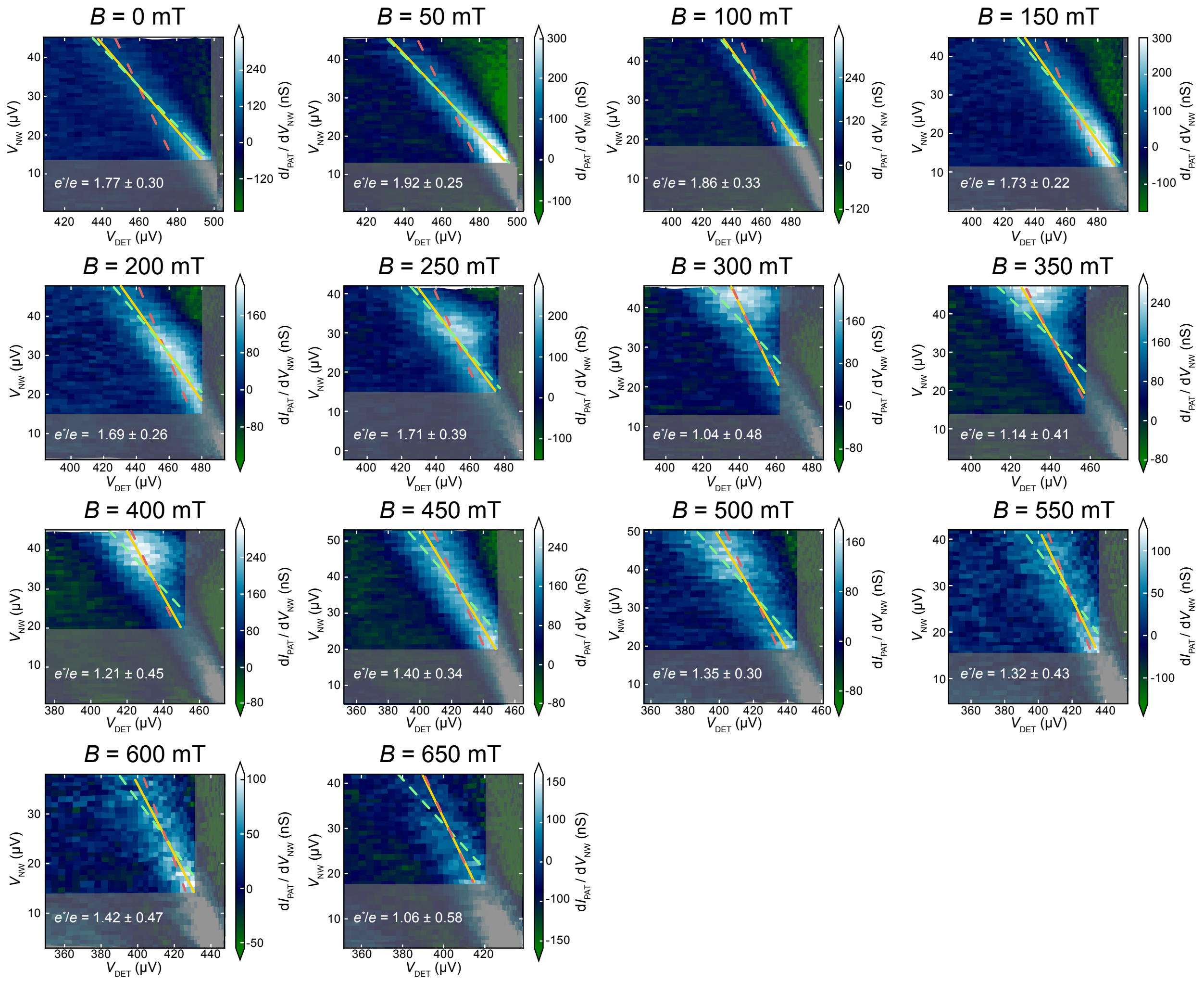

Supplementary Figure 3: **Complete magnetic field dependence for device NW1.** Differential transconductance $dI_{\mathrm{PAT}}\,/\,dV_{\mathrm{NW}}$ as a function of V_{NW} and V_{DET} for device NW1 at magnetic fields ranging between 0 and 650 mT. A linear fit $e^{\star}V_{\mathrm{NW}} = 2\Delta - eV_{\mathrm{DET}}$ through these peaks is shown as an orange line. Dashed green and red lines show linear fits with a fixed slope corresponding to $e^{\star} = 2e$ and $e^{\star} = e$, respectively. The shaded regions represent regimes where the fit of the transconductance peaks is not reliable either due to the phase diffusion regime at low V_{NW}, or due to high detector conductance at high V_{DET} values.

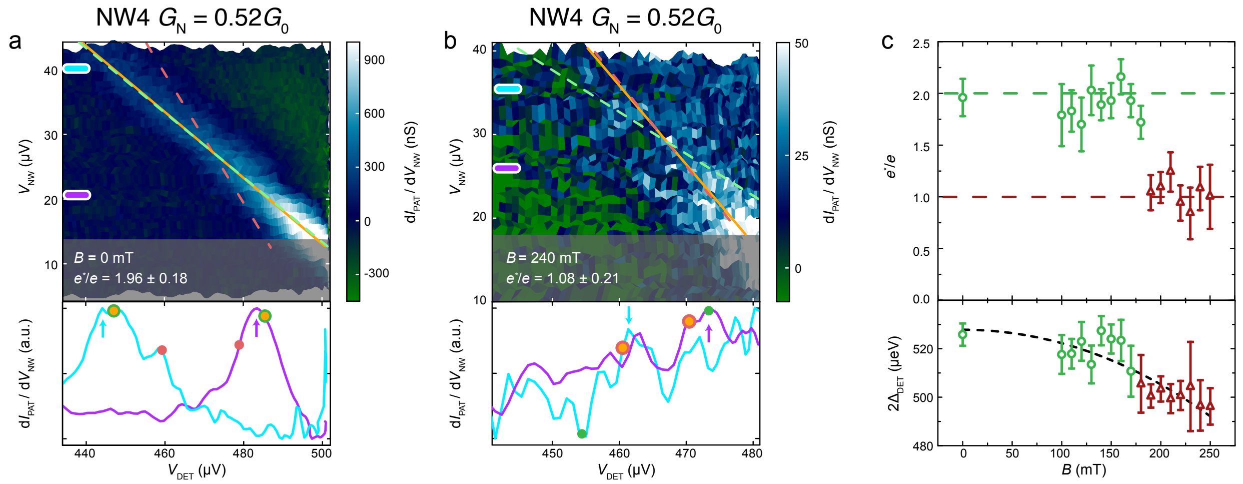

Supplementary Figure 4: **Magnetic field evolution of $e^\star$ for device NW4.** Differential transconductance $dI_{\text{PAT}}\,/\,dV_{\text{NW}}$ as a function of V_{NW} and V_{DET} for device NW4 at **(a)** $B = 0\,\text{mT}$ and **(b)** $B = 240\,\text{mT}$. A linear fit $e^\star V_{\text{NW}} = 2\Delta - eV_{\text{DET}}$ through these peaks is shown as an orange line. Dashed green and red lines show linear fits with a fixed slope corresponding to $e^\star = 2e$ and $e^\star = e$ respectively. The shaded regions represent regimes where the fit on the transconductance peak is not reliable. Normalised and smoothed horizontal linecuts are plotted at two V_{NW} values, where arrows show the position of the extracted peaks and orange, green and red dots denote the position of the best fit, the $e^\star = 2e$ fit and the $e^\star = e$ fit, respectively. **(c)** The effective charge $e^\star$ and Δ_{DET} extracted from the linear fit as a function of magnetic field. The transition from to 2π- to 4π-periodic Josephson radiation occurs at B $\approx 175\,\text{mT}$.

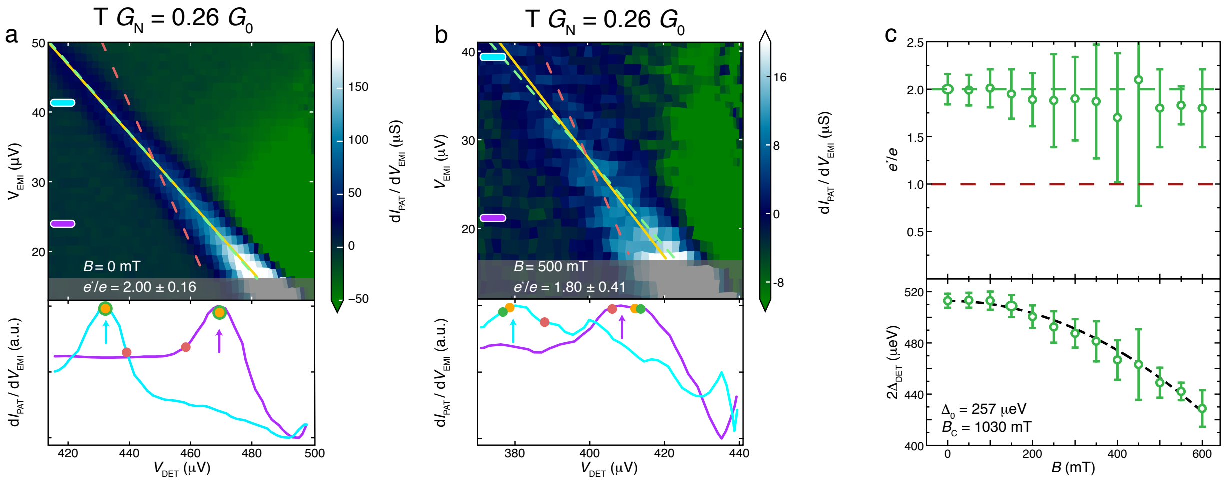

Supplementary Figure 5: **Magnetic field evolution of $e^\star$ for the tunnel junction device, T.** Differential transconductance $dI_{\mathrm{PAT}}\,/\,dV_{\mathrm{EMI}}$ as a function of V_{EMI} and V_{DET} for device T at **(a)** $B = 0\,\mathrm{mT}$ and **(b)** $B = 500\,\mathrm{mT}$. A linear fit $e^\star V_{\mathrm{EMI}} = 2\Delta - eV_{\mathrm{DET}}$ through these peaks is shown as an orange line. Dashed green and red lines show linear fits with a fixed slope corresponding to $e^\star = 2e$ and $e^\star = e$ respectively. The shaded regions represent regimes where the fit on the transconductance peak is not reliable. Normalised and smoothed horizontal linecuts are plotted at two V_{EMI} values, where arrows show the position of the extracted peaks and orange, green and red dots denote the position of the best fit, the $e^\star = 2e$ fit and the $e^\star = e$ fit, respectively. **(c)** The effective charge $e^\star$ and Δ_{DET} extracted from the linear fit as a function of magnetic field. No transition from to 2π- to 4π-periodic Josephson radiation occurs.

SUPPLEMENTARY TABLES

Parameter	NW1	NW2	NW3	NW4
Nanowire channel length (nm)	139	86	131	271
Total length between contacts (µm)	6.49	5.02	2.55	4.06
Detector tunnel junction area (µm^2)	0.040	0.014	0.025	0.024
Detector linear conductance, $G_{\mathrm{N,DET}}$ (G_0)	1.24	0.55	0.79	0.86
SQUID effective area, A_{SQUID} (µm^2)	5.34	3.62	5.37	5.49
Zero field detector gap, Δ_{DET} (µeV)	258	265	252	262
Detector critical field, $B_{\mathrm{C,DET}}$ (mT)	1180	670	1000	1070

Supplementary Table 1: Geometric and electrical parameters of the devices with a nanowire junction.

	NW1	NW2	NW3A	NW3B	NW4
Upper gate voltage, $V_{\mathrm{g}}^{\mathrm{upper}}$ (V)	-4.0	-0.95	-1.8	-2.22	1.75
Middle gate voltage, $V_{\mathrm{g}}^{\mathrm{middle}}$ (V)	-7.25	2.5	0.0	0.0	0.87
Lower gate voltage, $V_{\mathrm{g}}^{\mathrm{lower}}$ (V)	-4.0	-0.95	-1.08	-1.825	1.75
Emitter linear conductance, G_{N} (G_0)	0.37	0.52	0.57	0.73	0.52
Nanowire induced gap, Δ_{NW} (µeV)	232	105	60	84	164
4π transition field, $B^{\star}$ (mT)	300	260	175	–	190
Estimated eV_1 (µeV)	368	71	30	13	111

Supplementary Table 2: Electrical parameters of the nanowire junctions, specific to the gate voltage values used.

Parameter	Value
Emitter tunnel junction area (μm^2)	0.027
Emitter linear conductance, G_N (G_0)	0.26
Zero field gap, $\Delta(B = 0)$ (μeV)	247
Zero field critical current, $I_\mathrm{C} = \pi\Delta_\mathrm{EMI}G_\mathrm{N}/2e$ (nA)	7.58
Emitter junction critical field, $B_\mathrm{C,EMI}$ (mT)	953
Detector tunnel junction area, A_DET (μm^2)	0.023
Detector conductance, $G_\mathrm{N,DET}$ (G_0)	0.26
SQUID effective area, A_SQUID (μm^2)	5.44
Zero field detector gap, Δ_DET (μeV)	257
Detector critical field, $B_\mathrm{C,DET}$ (mT)	1030

Supplementary Table 3: Geometric and electrical parameters of device T with a superconducting tunnel junction as emitter, used for calibration purposes.

SUPPLEMENTARY NOTE 1: DETERMINATION OF THE SUPERCONDUCTING GAPS

This section describes the methods used to determine the superconducting gap in the nanowire and the detector. $2\Delta_{\mathrm{NW}}$ is inferred from the position of the coherence peaks in the nanowire differential conductance observed at the largest V_{NW}, as shown in Supplementary Figure 6a. The position of these peaks is comparable at both negative and positive bias voltages, and these values are consistent with the additional MAR features visible at fractions of $2\Delta_{\mathrm{NW}}$. The evolution of this gap with field for device NW1 is presented in Supplementary Figure 6b. Consistently with earlier tunneling spectroscopy experiments on similar devices [1–4] and with the theoretical literature [5–8], no gap closing is observed.

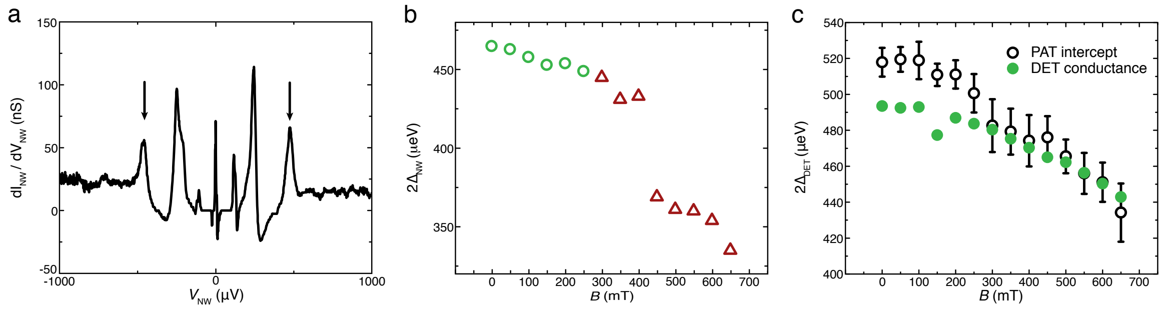

Supplementary Figure 6: **Identification of the superconducting gaps.** **(a)** Nanowire differential conductance ($\mathrm{d}I_{\mathrm{NW}}/dV_{\mathrm{NW}}$) as a function of nanowire voltage V_{NW} at B = 0 mT for device NW1. The coherence peaks at $2\Delta_{\mathrm{NW}}$ are identified by black arrows and are located at ± 465 μeV. The large peak at zero-bias arises from the supercurrent and the additional peaks are associated with multiple Andreev reflections (MAR). **(b)** Nanowire induced gap ($2\Delta_{\mathrm{NW}}$) as a function of magnetic field. Gaps obtained in the 2π- and the 4π-periodic regimes are shown as green circles and red triangles, respectively. **(c)** Al/AlO$_x$/Al detector gap ($2\Delta_{\mathrm{DET}}$) as a function of magnetic field for device NW1. The gap value is either obtained from the intercept of the fitted PAT data (open black circles, same as Fig. 2d) or from the raw IV-trace of the detector (solid green circles).

As shown in Supplementary Figure 6c, the gap value of the SIS detector can be obtained either from the intercept of the fitted PAT data, as extracted in Fig. 2d, or from the raw IV-trace of the detector (Fig. 1h). Due to the limited range of the detector IV-trace, the

gap is determined by the point at which the derivative of the detector IV-trace reaches 500 μS. The two methods agree well at magnetic fields larger than 250 mT. The discrepancy at lower fields is attributed to local heating effects at finite bias across the superconductor, which artificially increases the derivative of the detector IV-trace near the gap edge. Due to the unreliability of the data extracted from the IV at low magnetic fields, the gap value extracted from the fitted PAT data has been reported in Supplementary Table 2.

SUPPLEMENTARY NOTE 2: PEAK FINDING AND FITTING

This section describes the methodology for finding the peaks of the PAT current transconductance, and subsequently fitting them to obtain the effective charge $e^{\star}$. An example of a typical transconductance scan in shown in Supplementary Figure 7a. For every V_{NW} trace taken during this scan (see Supplementary Figure 7b for a typical line trace), the data is interpolated into 100 evenly spaced points. This interpolated data is then smoothed using a 2nd order polynomial Savitzky-Golay filter with a dynamical window length ranging between 5 and 21 points (red line in Supplementary Figure 7b). The window length scales linearly, with the larger window size being used at larger V_{NW}. The f^{-2} dependence of the PAT signal [9] results in a decreased signal-to-noise ratio at large V_{NW}, making additional smoothing desirable. Next, the peak from this smoothed curve is extracted. The data is then further smoothed using a 51 points dynamical window (not shown in Supplementary Figure 7b), and the first relative minimum reached on each side of the peak is used to define the peak minimum. The half-width at half-maximum on each side of the peak are then averaged, and used as the uncertainty on the peak position.

The peak finding algorithm initially finds the peak for the lowest V_{NW} trace. To ensure that neither the current associated with quasiparticle tunneling at high V_{NW} values near the gap edge nor noise signals far away from the peak are inadvertently selected, all subsequent peaks are required to be located at a position in V_{DET} within a moving window $V_{\mathrm{DET}}^{\mathrm{last}} - 28\,\mu\mathrm{V} \leq V_{\mathrm{DET}}^{\mathrm{new}} \leq V_{\mathrm{DET}}^{\mathrm{last}} + 5\,\mu\mathrm{V}$.

Once the transconductance peaks are obtained, they are fitted using a Monte-Carlo bootstrap method with 10000 iterations [10]. For every iteration, the peak position of each V_{NW} trace is randomly determined from a Gaussian distribution with the average and the deviation given by the original peak position and uncertainty. These generated peaks are then linearly fitted and their slope and intercept are collected. The reported slope and intercept are given by the average results obtained over the 10000 iterations, and the uncertainty is given by their standard deviation.

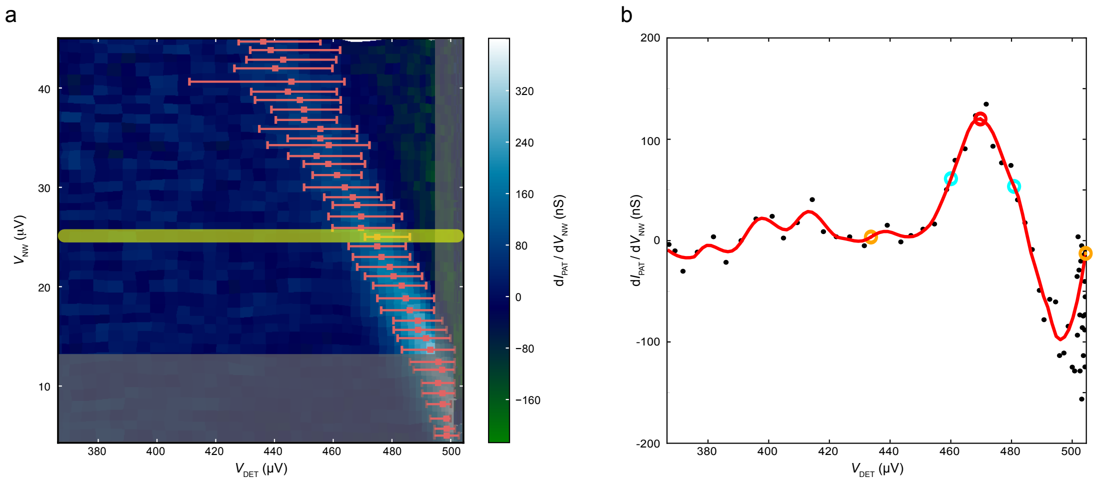

Supplementary Figure 7: **Peak finding algorithm.** **(a)** Transconductance of the PAT current as a function of the bias across the NW junction and the detector for device NW1 at B = 0 mT. The position of the transconductance peak for each V_{NW} trace, with its uncertainty, is shown as a red dot with error bars. The shaded regions represent regimes where the position of the transconductance peak is shifted due to the large sub-gap current in the detector (x-axis) and/or due to the presence of phase-diffusion in the nanowire junction (y-axis). **(b)** Typical processed line-cut used to extract the transconductance peak. This line-cut corresponds to the yellow line in panel **(a)**. The maximum (red dot) is extracted from the smoothed curve (shown in red) and corresponds to the red dots in panel **(a)**. The estimated peak minimums (orange dots) and the corresponding half-maximum values (cyan dots, corresponding to the error bars in panel **(a)** are also shown.

The fitting procedure is carried out over a subset of the raw dataset. The lower bound of the fitting range is set so as to exclude two phenomena causing the extracted peaks to be improperly linked to the Josephson radiation, as inferred from Eq. (3). The first phenomenon stems from the assumption that the detector IV characteristics is ideal, *i.e.* that zero subgap current is present at detector voltages lower than the superconducting gap. While this assumption is realistic at V_{DET} values sufficiently far from the superconducting gap, it breaks down as V_{DET} is increased, and Eq. (2) is not expected to remain valid at these voltages. The breakdown point has been empirically determined in a trivial device (at B = 0 mT), and is reached when the derivative of the detector IV at V_{NW} = 0 μV is greater than 10 μS (see Supplementary Figure 8a). This regime is represented by the vertical

gray-shaded area in Supplementary Figure 7a.

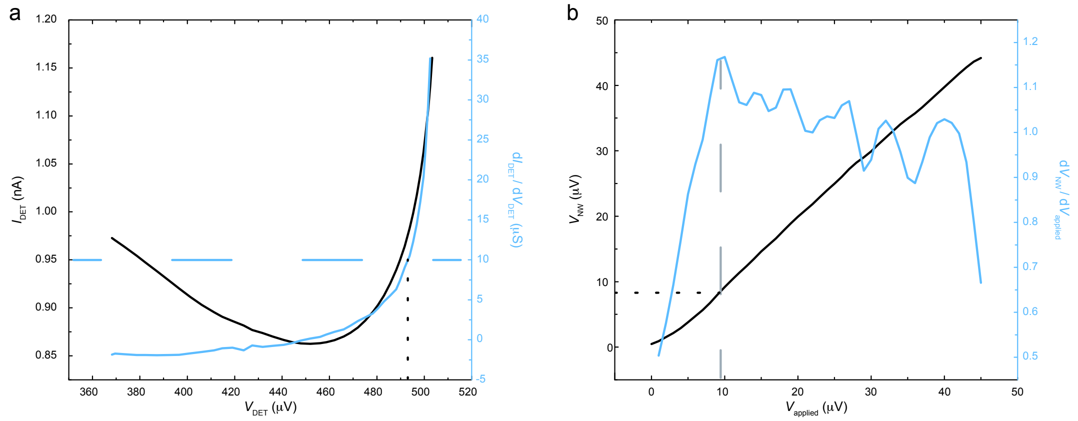

Supplementary Figure 8: **Experimental determination of the reliable biasing regions. (a)** Detector current (left axis) and derivative of the detector current (right axis) as a function of the detector voltage. The detector voltage cut-off (black dotted line) is determined by the value at which the derivative of the detector current at $V_{\text{NW}} = 0$ µV reaches 10 µS (blue dashed line). **(b)** Measured voltage across the nanowire junction (left axis) and derivative of the measured voltage across the nanowire junction (right axis) as a function of the applied voltage across the nanowire side of the circuit. The nanowire voltage cutoff is 5 µV larger than the value of the first peak in the derivative of the measured nanowire voltage (gray dashed line). All data was taken on device NW1 at $B = 0$.

Phase diffusion in the nanowire junction can also induce a discrepancy between the position of the transconductance peak and the Josephson radiation extracted using Eq. (3). The dynamics of this phenomenon are presented in details later in this supplement. The phase-diffusion regime is characterized by a non-linearity between the applied voltage and the measured voltage across the nanowire junction. Experimentally, we define this regime by locating the peak in the (low-pass filtered) derivative of the measured voltage with respect to the applied voltage (see Supplementary Figure 8b). Only data obtained at voltages 5 µV larger than this peak are considered reliable. The excluded phase-diffusive region occurs at V_{NW} values located in the horizontal gray-shaded area in Supplementary Figure 7a.

SUPPLEMENTARY NOTE 3: THEORY

Probabilistic model of voltage biased Majorana Josephson junction

For simplicity, we consider a single conduction channel through the proximitized nanowire. In the topological regime, four Majorana states emerge; two near the junction, and two at the far ends of the wire. Taking into account the overlaps between these Majorana states [11], one obtains two many-body levels of each parity, with energies of the form

$$E_{\pm,o/e}(\phi) = \pm\sqrt{\varepsilon_{M,o/e}^2 + \epsilon^2\cos^2(\phi/2)}\,. \tag{1}$$

Here ϕ is the gauge-invariant phase difference across the junction, ϵ is a typical coupling energy of the Majorana states near the junction, and $\varepsilon_{M,o/e}$ is set by the couplings of these interior Majorana states to the ones on the wire ends. The phase dependence exhibits avoided level-crossings at $\phi = (2n+1)\pi$, with minimum energy splitting $\varepsilon_{M,o/e}$ between the upper and lower branches of the odd or even parity states respectively. We make the simplifying assumption that the odd and even states have the same energy, $E_\pm(\phi)$ (i.e. that $\varepsilon_{M,o/e} = \varepsilon_M$). The supercurrent in the lower (ground state; $-$) or upper (excited; $+$) branch is

$$I_\pm(\phi) = \frac{2e}{\hbar}\frac{\partial E_\pm}{\partial\phi} \approx \mp I_m\,\mathrm{sgn}[\cos(\phi/2)]\sin(\phi/2)\,, \tag{2}$$

where the last formula holds in the limit $\varepsilon_M \to 0$, with $I_m = e\epsilon/\hbar$. When the junction is biased by a DC voltage V, the phase difference ϕ winds linearly with time, $\dot{\phi} = (2e/\hbar)V$. For sufficiently small bias, the system evolves adiabatically (entirely in the lower branch at $T = 0$) and the usual Josephson effect results: the supercurrent oscillates at the Josephson frequency $\omega_J = (2e/\hbar)V$. Larger bias voltages can induce Landau-Zener (LZ) tunneling [12, 13] between branches at $\phi = (2n+1)\pi$. The system can also tunnel from the upper branch to the continuum at $\phi = 2n\pi$, effectively emitting a quasiparticle and relaxing to the lower branch in the process. In addition, the quantum dynamics of the junction are affected by thermal relaxation, dephasing, and quasiparticle poisoning processes.

The appropriate model for the dynamics of the junction depends on the nature and strength of the coupling to the external circuit. We assume that this coupling is strong, and that the external circuit, which can be described classically, frequently measures the current through the junction. As a result of such a measurement, the junction collapses into a state

of definite current, i.e. into either the upper or lower branch, $E_{\pm}(\phi)$. Motivated by this reasoning, we describe the instantaneous state of the junction by a random variable $\zeta = \pm 1$, which labels the branches. LZ tunneling between branches is described by the transition probability

$$P(\zeta \to -\zeta) = p_0(V) = \exp(-V_0/V) \tag{3}$$

whenever ϕ crosses $(2n+1)\pi$, where $V_0 \approx 4\pi\varepsilon_M^2/e\epsilon$ is the LZ voltage scale and V is the instantaneous voltage across the junction. Tunneling to the continuum is described by a transition probability of the same form [14, 15],

$$P(\zeta = +1 \to \zeta = -1) = p_1(V) = \exp(-V_1/V) \tag{4}$$

whenever ϕ crosses $2n\pi$, where $V_1 = 2\pi\Delta_{\rm NW}(1-\sqrt{\tau})^2/e\sqrt{\tau}$ is another voltage scale. We ignore thermal relaxation of the junction state, since the associated timescale is large relative to an oscillation period. Quasiparticle poisoning is described by a constant rate Γ_q for transitions from $\zeta \to -\zeta$ (independent of ϕ). Although these approximations are drastic, we expect our results to be qualitatively correct as long as decoherence is fast enough relative to other timescales. The opposite limit of very slow decoherence is discussed in [11]. Effective discrete Markov models such as ours have been used to describe the non-adiabatic dynamics of Majorana bound states in the past, for instance by [15].

Power spectrum for fixed junction voltage (no external circuit)

We first study the dynamics of our simplified model when the Majorana junction is biased by a constant external voltage $V > 0$, so that the phase difference across the junction increases linearly with time; $\phi(t) = \phi_0 + (2e/\hbar)Vt$. In this case, one can obtain an analytical result for the power spectral density of the supercurrent noise. The supercurrent branch of the system changes randomly due to LZ tunneling every time ϕ passes an odd multiple of π, but we temporarily ignore tunneling to the continuum.

The supercurrent power spectral density (PSD) is defined as

$$S_I(\omega; V, V_0) = \lim_{t_f \to \infty} \frac{1}{t_f} \int_0^{t_f} dt \int_0^{t_f} dt' \, \langle I(t) I(t') \rangle \, e^{i\omega(t-t')} \tag{5}$$

where $I(t)$, the supercurrent at time t, is a random variable due to LZ tunneling with

associated voltage scale V_0. We show later in this Supplement that

$$S_I(\omega; V, V_0) = \left(\frac{\hbar I_m^2}{eV}\right) F\left(\frac{\hbar\omega}{2eV}, \frac{V}{V_0}\right) \tag{6}$$

where $F(x, y)$ is the scaled PSD,

$$F(x, y) = \left[\frac{x^2(1 + \cos 2\pi x)}{\pi(x^2 - 1/4)^2}\right] \left[\frac{1 - (1 - 2e^{-1/y})^2}{1 + (1 - 2e^{-1/y})^2 - 2(1 - 2e^{-1/y}) \cos 2\pi x}\right]. \tag{7}$$

Note that x is simply the frequency ω measured in units of the Josephson frequency $\omega_J = (2e/\hbar)V$. The function $F(x, y)$ is plotted in Supplementary Figure 9 and Supplementary Figure 10. We draw attention to the fact that, for V between $\sim V_0$ and $\sim 2V_0$, the spectral density is broad but has a maximum at a frequency ω_* intermediate between ω_J and $\omega_J/2$.

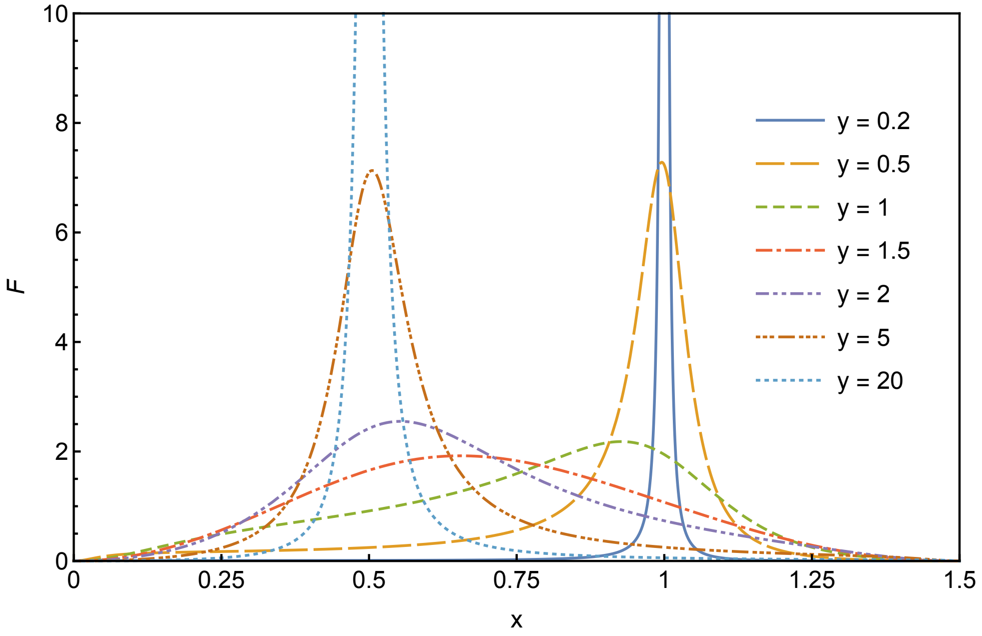

Supplementary Figure 9: **Scaled spectral density.** The scaled spectral density $F(x, y)$, defined in Eq. (7), is plotted versus x for specified values of y. The variable x is frequency measured in units of Josephson frequency $\omega_J = (2e/\hbar)V$, while $y = V/V_0$ is the ratio of the bias voltage to the characteristic LZ tunneling voltage. At small bias $V/V_0 \ll 1$, the PSD exhibits a sharp peak at $\omega = \omega_J$. Over an intermediate bias range $V/V_0 \sim 1 - 2$, the PSD broadens, with the maximum gradually moving from $\omega = \omega_J$ to $\omega = \omega_J/2$. Finally, for large bias $V/V_0 \gg 1$, the PSD becomes sharply peaked at $\omega = \omega_J/2$. Note that the peaks for $y = 0.2$ and 20 extend far beyond the range of the plot.

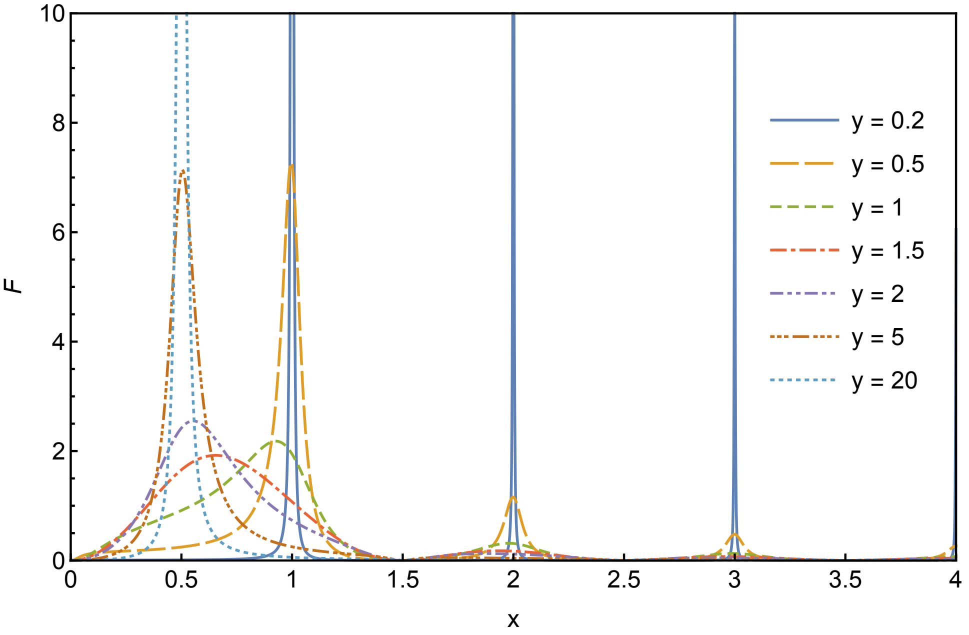

Supplementary Figure 10: **Scaled spectral density over a larger range.** Same linecuts of $F(x, y)$ as in Supplementary Figure 9, but plotted over a larger range of x. The PSD is clearly peaked at all integer multiples of the Josephson frequency, $\omega = n\omega_J$. However, the weight of these peaks decreases rapidly with both increasing n and increasing y; even the $n = 2$ peak is negligible by $y = V/V_0 \approx 1.5$. The dependence on y is understood as follows: for $V \ll V_0$, the dynamics are essentially adiabatic, and the peaks at $\omega = n\omega_J$ come from higher harmonics in the expansion of the supercurrent $I(\phi) \sim I_m \,\mathrm{sgn}[\cos(\phi/2)] \sin(\phi/2)$, which changes abruptly from $+I_m$ to $-I_m$ as ϕ crosses π. When $V \geq V_0$, on the other hand, LZ transitions between the branches smooth the effective supercurrent-phase relation and wash out these higher harmonics. In the limit $V \gg V_0$, the system tracks a smooth 4π-periodic $I(\phi) \sim \sin(\phi/2)$, leading to the sharp peak at $\omega = \omega_J/2$, without any higher harmonics.

The above results are valid in the regime $V \ll V_1$, where V_1 is the voltage scale for tunneling to the continuum (Eq. (4)). The effect of finite V_1 on the dynamics of a perfectly 4π-periodic bound state (corresponding to $V_0 = 0$) has been studied in detail by Houzet et al. [15]. Eq. (14) in their paper gives the PSD obtained by solving an effective discrete Markov model very similar to ours, but with $V_0 = 0$ and V_1 finite. Their result may be compared with our Eqs. (6) and (7), which is for V_0 nonzero and $V_1 \to \infty$. Qualitatively,

large but finite $V_1 > V$ broadens the $\omega_J/2$ peak in Figs. 9 and 10, as it provides an additional decay channel for the effective 4π-periodic state. When $V \gtrsim V_1$, the $\omega_J/2$ peak is strongly suppressed because the junction transitions out of the upper branch at $\phi = 2n\pi$ with high probability. Therefore, the ideal PSD $S_I(\omega)$ exhibits a clear peak at half the Josephson frequency only in the voltage range $2V_0 \lesssim V < V_1$.

Modeling the junction environment: equivalent circuit

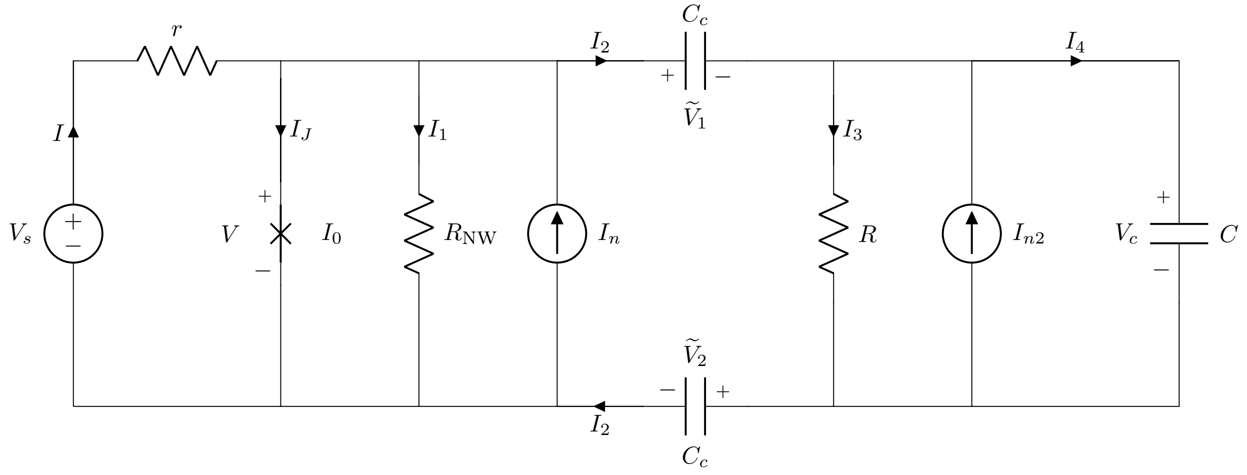

Supplementary Figure 11: **Circuit diagram.** Physical circuit parameters are: DC source voltage V_s, resistances r, R_{NW}, R, and capacitances C, C_c. $V, V_c, \widetilde{V}_1, \widetilde{V}_2$ are time-dependent voltages across the indicated circuit elements. The current source I_{n2} describes Johnson noise of the resistance R; current source I_n describes the combined Johnson-Nyquist noise of resistances R_{NW} and r. Note that here, R_{NW} represents the sub-gap resistance in the relevant voltage range, and is in general larger than the normal-state resistance of the nanowire.

We model the dynamics of the nanowire Josephson junction, along with its RF environment, via the following equivalent circuit (this corresponds to the left half of Fig. 1b in the main text), which includes Johnson-Nyquist noise current sources for the various resistances. The detector part of the circuit (right half of Fig. 1b) is not modeled explicitly; the capacitive coupling via C_c of the emitter and detector decouple them at low-frequencies, and we assume that any high-frequency feedback from the detector can be absorbed into a renormalization of the effective environment capacitance C. The measured noise power

spectral density at the detector is approximated by the PSD of the voltage $V_c(t)$ across the capacitor C.

First consider a standard Josephson tunnel junction in the non-topological regime (we will treat the topological case in the next section). The phase difference $\phi(t)$ across the ideal Josephson junction element obeys

$$\frac{d\phi}{dt} = \frac{2e}{\hbar} V(t) \tag{8}$$

and the supercurrent through the junction is given by

$$I_J(t) = I_0 \sin\phi(t)\,. \tag{9}$$

The current sources I_n and I_{n2} represent white Gaussian noise with zero mean and autocorrelation

$$\langle I_n(t_2) I_n(t_1)\rangle = 2k_B T \left(\frac{1}{r} + \frac{1}{R_{\mathrm{NW}}}\right)\delta(t_2 - t_1)\,, \qquad \langle I_{n2}(t_2) I_{n2}(t_1)\rangle = \frac{2k_B T}{R}\delta(t_2 - t_1) \tag{10}$$

respectively, where T is the effective noise temperature (assumed to be the same for all resistors). Note: if one introduces independent noise current sources I_{na}, I_{nb} for resistors r and R_{NW}, only the sum $I_{na} + I_{nb}$ appears in the equations of motion, and $I_n = I_{na} + I_{nb}$ has the specified autocorrelation function.

The quantities of interest are the instantaneous current $I(t)$, voltage $V(t)$ across the junction, and voltage $V_c(t)$ across capacitor C. It is convenient to describe the circuit

dynamics in terms of dimensionless variables. If we define

$$
\begin{aligned}
R_{\text{eff}} &= (1/r + 1/R_{\text{NW}})^{-1} \\
\omega_0 &= 2eI_0 R_{\text{eff}}/\hbar \\
\tau &= \omega_0 t \\
v &= V/R_{\text{eff}} I_0 \\
v_c &= V_c/RI_0 \\
v_s &= V_s/rI_0 \\
i_n &= I_n/I_0 \\
i_{n2} &= I_{n2}/I_0 \\
Q^2 &= \omega_0 R_{\text{eff}} C = 2eI_0 R_{\text{eff}}^2 C/\hbar \\
\alpha &= R_{\text{eff}}/R \\
\gamma &= 2C/C_c \\
\Gamma &= 2ek_B T/\hbar I_0,
\end{aligned}
\tag{11}
$$

then the equations of motion take the form (derivations are shown later in this Supplement)

$$
\begin{aligned}
\frac{d\phi}{d\tau} &= v\,, \\
\frac{dv}{d\tau} &= Q^{-2}\Big[(1+\gamma)(v_s - v - \sin\phi + i_n) - v_c + i_{n2}\Big]\,, \\
\frac{dv_c}{d\tau} &= \alpha Q^{-2}\Big[v_s - v - v_c - \sin\phi + i_n + i_{n2}\Big]\,,
\end{aligned}
\tag{12}
$$

where i_n, i_{n2} represent white Gaussian noise with zero mean and autocorrelation

$$
\begin{aligned}
\langle i_n(\tau_2) i_n(\tau_1)\rangle &= 2\Gamma\delta(\tau_2 - \tau_1)\,, \\
\langle i_{n2}(\tau_2) i_{n2}(\tau_1)\rangle &= 2\alpha\Gamma\delta(\tau_2 - \tau_1)\,.
\end{aligned}
\tag{13}
$$

The system of stochastic differential equations Eq. (12) describes a nonlinear stochastic process in the three dimensional state space spanned by the variables ϕ, v and v_c. We can gain insights into the properties of typical solutions by studying the attractors of the underlying deterministic process,

$$
\begin{aligned}
\frac{d\phi}{d\tau} &= v\,, \\
\frac{dv}{d\tau} &= Q^{-2}\Big[(1+\gamma)(v_s - v - \sin\phi) - v_c\Big]\,, \\
\frac{dv_c}{d\tau} &= \alpha Q^{-2}\Big[v_s - v - v_c - \sin\phi\Big]\,,
\end{aligned}
\tag{14}
$$

i.e. the zero temperature limit of Eq. (12). For given parameters (α, γ, Q, v_s), the solution to Eq. (14) with initial condition (ϕ, v, v_c) defines a map $f_\tau : (\phi, v, v_c) \mapsto (\phi(\tau), v(\tau), v_c(\tau))$. We identify the attractors and their basins of attraction by numerically evaluating f_τ for large τ. The results are plotted in Figs. 12 and 13 for various parameter values. As in the simpler RCSJ model, the attractors of Eq. (14) come in two varieties: zero-voltage attractors, in which $v = v_c = 0$ and $\phi = \phi_*$ is constant, and a nonzero-voltage attractor, in which $v > 0$ and the phase winds. We will call these 0 and 1 states for brevity.

The low-temperature, noisy dynamics of the system can be understood in terms of diffusive motion around an attractor in state space, together with intermittent noise-induced transitions between the attractors [16]. We identify a thermally activated phase diffusion regime at low bias voltages, $v_s < v_{s1}$, in which the measured average voltage $\bar{v}$ is entirely due to the difference in transition rates for thermally activated $\phi_* \to \phi_* + 2\pi$ and $\phi_* \to \phi_* - 2\pi$ processes. At intermediate bias, $v_{s1} < v_s < v_{s2}$, the dynamics is more complicated, with the possibility of thermally activated transitions between coexisting 0 and 1 states. In this switching regime, the measured $\bar{v}$ can be interpreted as a weighted mean of the average voltage in the 1 state and the phase diffusion voltage. Finally, for $v_s > v_{s2}$, the dynamics reduces to small thermal fluctuations around the 1 state.

We conclude from this analysis that, when $v_s < v_{s2}$, the frequency of radiation emitted by the junction is not simply related to the Josephson frequency $\omega_J = (2e/\hbar)\overline{V}$. On the other hand, when $v_s > v_{s2}$, the dynamics of the external circuit does not play a major role, and the emitted radiation faithfully represents the dynamics of the junction (we verify this assertion by numerically solving the circuit equations—see Fig. 4 in the main text and Supplementary Figure 17 below).

In the $\Gamma \ll 1$ limit, $v_{s2} \approx 1$, since this is the bias voltage at which the 0 state attractors vanish. For parameter values $Q \sim 1.1$, $\alpha \sim 4.6$, and $\gamma \sim 0.05$, we find that $v_{s1} \approx 0.58$ (see Supplementary Figure 12). For larger Γ, the threshold bias $v_{s2}(\Gamma)$ may be estimated as the value of v_s above which $d\bar{v}/dv_s \approx 1$. In general, $v_{s2}(\Gamma) > v_{s2}$.

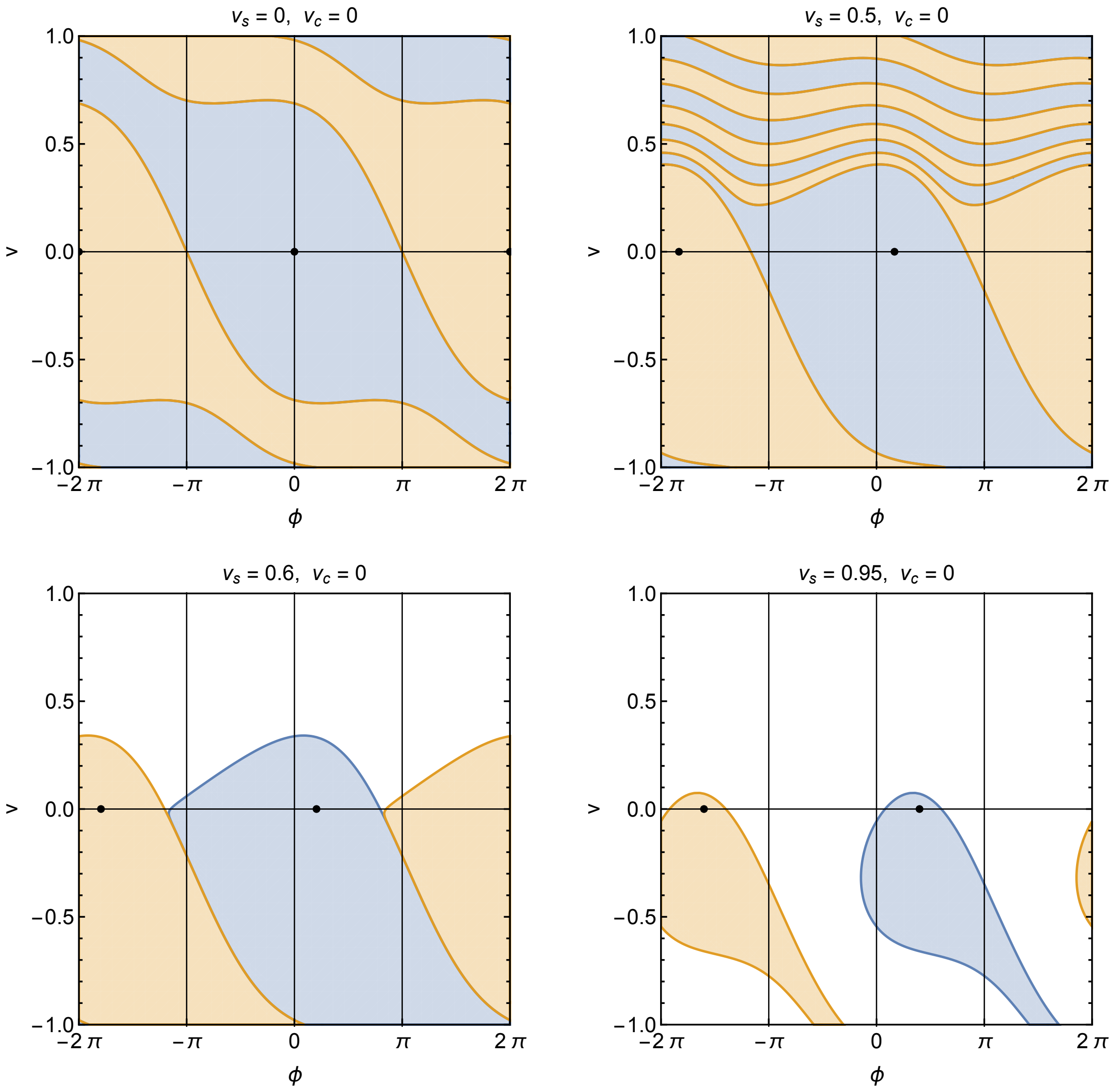

Supplementary Figure 12: **Basins of attraction in the non-topological case.** Basins of attraction of Eq. (14) on a $v_c = 0$ slice through state space, for various values of dimensionless bias voltage v_s. Other dimensionless parameters are fixed at $Q = 1.1, \alpha = 4.6, \gamma = 0.05$ (these are the approximate experimental values). The blue and orange regions are stable manifolds of 0 state attractors; every point in such a region flows to the same 0 state. Two 0 state attractors are shown (black dots). The white region is the stable manifold of the 1 state; every point in this region flows to the same periodic 1 state trajectory (not shown here). Note that the state space and all attractors are 2π-periodic in ϕ.

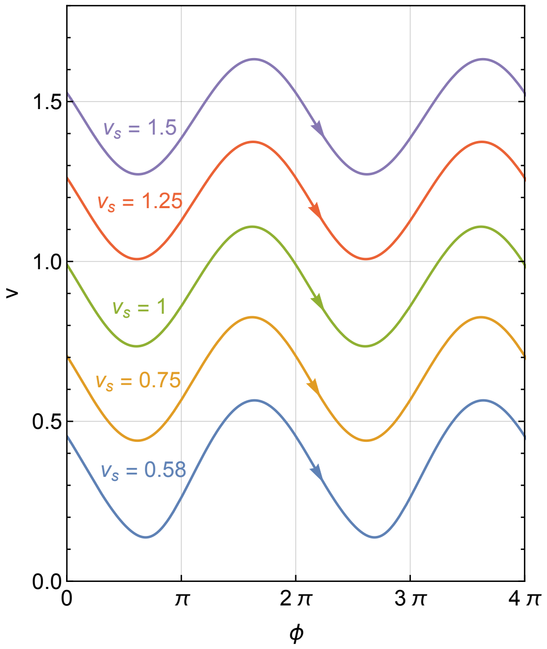

Supplementary Figure 13: **Non-topological 1 state attractor.** Trajectories of the 1 state attractor for various values of dimensionless bias voltage v_s, in the non-topological case Eq. (14). The two panels show projections of the attractors onto the (ϕ, v) and (v_c, v) planes, respectively. As is clear from the left panel, ϕ increases by 2π over an orbital period. Other dimensionless parameters are fixed at $Q = 1.1$, $\alpha = 4.6$, and $\gamma = 0.05$ (these are the approximate experimental values). Note that, for these parameter values, the 1 state is only stable for $v_s \gtrsim 0.58$ (for smaller v_s, all initial points flow to a 0 state).

Circuit equations in the topological regime

In the topological regime, the Josephson current relation Eq. (9) is replaced, in our model of the junction, by

$$I_J(t) = I_m\,\zeta(t)\,\mathrm{sgn}[\cos(\phi(t)/2)]\,\sin(\phi(t)/2)\,. \tag{15}$$

Here $\zeta = \pm 1$ is a random variable that accounts for LZ tunneling, as well as tunneling to the continuum, as discussed previously. The critical current I_m of the Majorana junction replaces I_0, the critical current of the trivial junction (the definitions in Eq. (11) are modified accordingly).

As a result of this replacement, the dimensionless circuit equations Eq. (12) are modified to

$$\begin{aligned}
\frac{d\phi}{d\tau} &= v\,,\\
\frac{dv}{d\tau} &= Q^{-2}\Big[(1+\gamma)(v_s - v - \zeta\,\mathrm{sgn}[\cos(\phi/2)]\sin(\phi/2) + i_n) - v_c + i_{n2}\Big]\,,\\
\frac{dv_c}{d\tau} &= \alpha Q^{-2}\Big[v_s - v - v_c - \zeta\,\mathrm{sgn}[\cos(\phi/2)]\sin(\phi/2) + i_n + i_{n2}\Big]\,.
\end{aligned} \tag{16}$$

As before, we may study the stable fixed points (0 state) and limit cycle (1 state) of an underlying deterministic process. There are two distinct deterministic limits: i) the junction evolves adiabatically, remaining always in its ground state. This corresponds to fixing $\zeta = 1$ in Eq. (16). In this case the attractors and their basins of attraction are qualitatively similar to those of the non-topological junction (Figs. 12 and 13). ii) the evolution is perfectly non-adiabatic. This corresponds to fixing $\zeta\,\mathrm{sgn}[\cos(\phi/2)] = 1$ in Eq. (16). An ideal topological junction (in which there is negligible hybridization between the Majorana states at the junction and those at the far ends of the wire) would be described by case ii) at any nonvanishing voltage.

The results for case ii) are plotted in Figs. 14 and 15. The analysis of the noisy dynamics in terms of phase-diffusion, switching, and running regimes applies here as well. In particular, when $v_s < v_{s2}$, it is possible for the junction to radiate at a frequency intermediate between $\omega_J/2$ and ω_J, where $\omega_J = (2e/\hbar)\overline{V}$ is the Josephson frequency, even when the junction bound state is perfectly 4π-periodic ($V_0 \to 0$ and $V_1 \to \infty$).

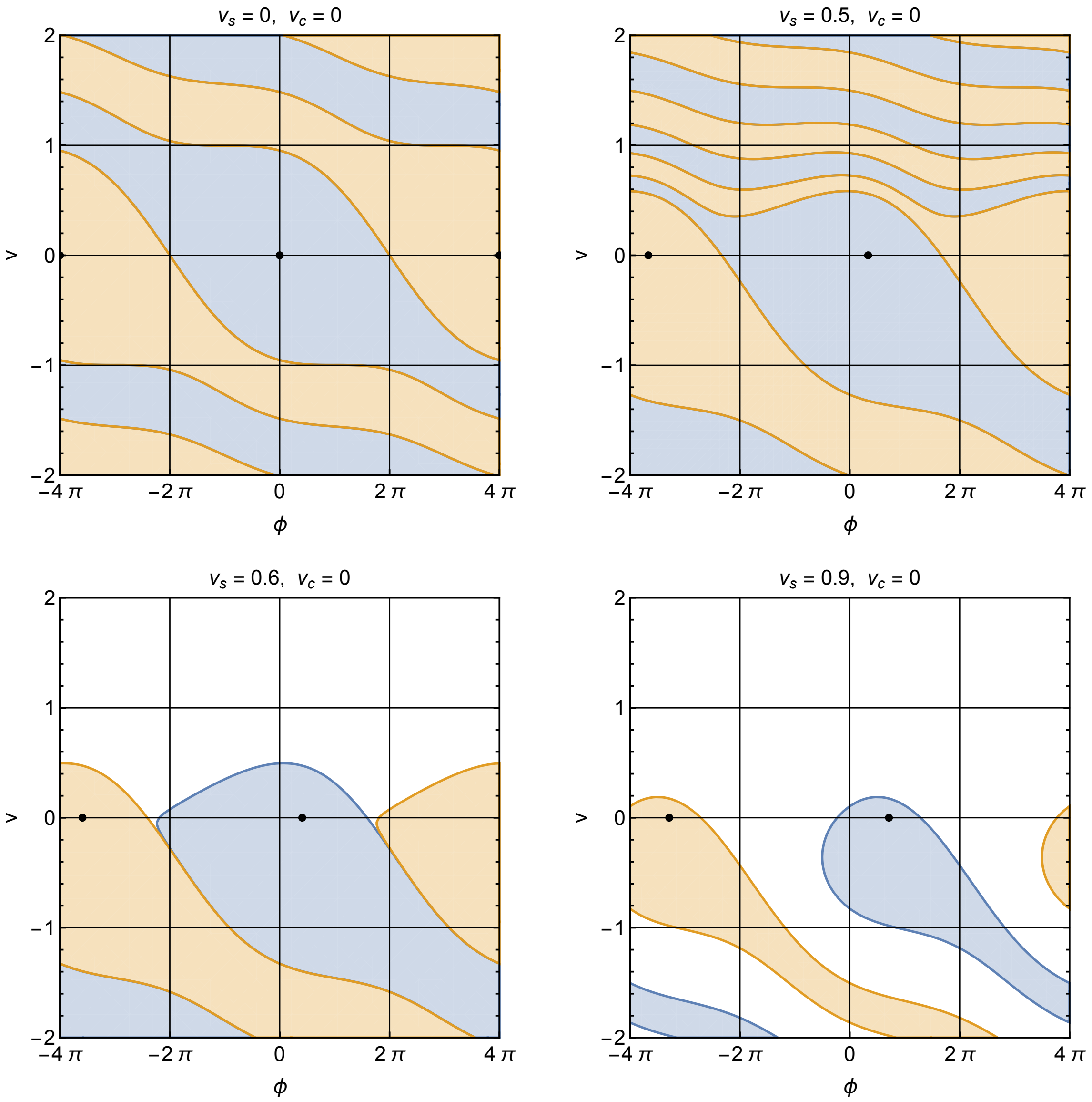

Supplementary Figure 14: **Basins of attraction in the topological case.** Basins of attraction of Eq. (16) with $\zeta = +1$ on a $v_c = 0$ slice through state space, for various values of dimensionless bias voltage v_s. Other dimensionless parameters are fixed at $Q = 1.1, \alpha = 4.6, \gamma = 0.05$ (these are the approximate experimental values). The blue and orange regions are stable manifolds of 0 state attractors; every point in such a region flows to the same 0 state. Two 0 state attractors are shown (black dots). The white region is the stable manifold of the 1 state; every point in this region flows to the same periodic 1 state trajectory (not shown here). Note that the state space and all attractors are 4π-periodic in ϕ.

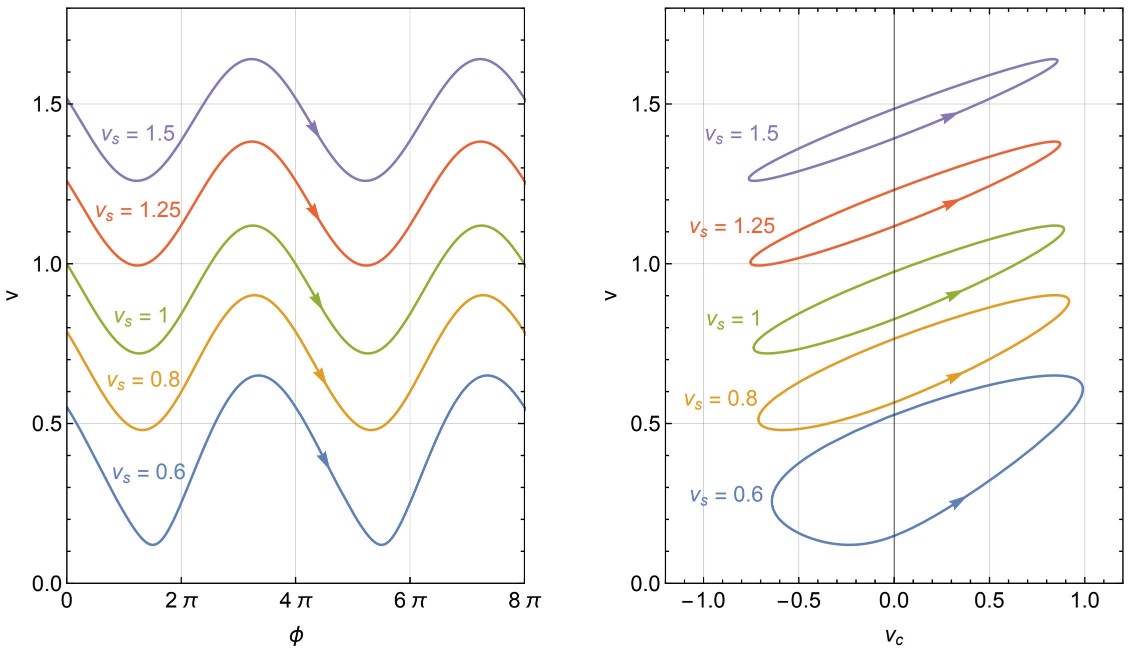

Supplementary Figure 15: **Topological 1 state attractor.** Trajectories of the 1 state attractor for various values of dimensionless bias voltage v_s, in the topological case (Eq. (16) with $\zeta = +1$). The two panels show projections of the attractors onto the (ϕ, v) and (v_c, v) planes, respectively. As is clear from the left panel, ϕ increases by 4π over an orbital period. Other dimensionless parameters are fixed at $Q = 1.1$, $\alpha = 4.6$, and $\gamma = 0.05$ (these are the approximate experimental values). Note that, for these parameter values, the 1 state is only stable for $v_s \gtrsim 0.6$ (for smaller v_s, all initial points flow to a 0 state).

Numerical solution of circuit equations

We numerically integrate the stochastic differential equations describing the circuit using the first-order Euler-Maruyama method [17]. The update rule with step size $\delta\tau$ is

$$\begin{aligned}
\phi_{j+1} &= \phi_j + v_j \delta\tau \,, \\
v_{j+1} &= v_j + Q^{-2}\Big[(1+\gamma)\,(v_s - v_j - \zeta_j s(\phi_j)) - (v_c)_j\Big]\delta\tau + Q^{-2}\sqrt{2\Gamma\delta\tau}\Big[(1+\gamma)X_{1,j} + \sqrt{\alpha}\,X_{2,j}\Big]\,, \\
(v_c)_{j+1} &= (v_c)_j + \alpha Q^{-2}\Big[v_s - v_j - (v_c)_j - \zeta_j s(\phi_j)\Big]\delta\tau + \alpha Q^{-2}\sqrt{2\Gamma\delta\tau}\Big[X_{1,j} + \sqrt{\alpha}\,X_{2,j}\Big]\,,
\end{aligned} \tag{17}$$

where $s(\phi) = \mathrm{sgn}[\cos(\phi/2)]\sin(\phi/2)$ for the topological junction, and where $X_{i,j}$ are independent identically distributed standard normal (mean 0 and variance 1) random variables.

The probabilistic update rule for $\zeta = \pm 1$ takes into account quasiparticle poisoning, LZ tunneling between branches, and tunneling to the continuum:

$$P(\zeta_{j+1} = \zeta_j) = \begin{cases} (1-q)(1-p_{0j}) + qp_{0j} & \text{if} \quad \cos(\phi_j/2)\cos(\phi_{j+1}/2) < 0\,, \\ (1-q)(1-p_{1j}) + qp_{1j} & \text{if} \quad \zeta_j = -1 \quad \text{and} \quad \sin(\phi_j/2)\sin(\phi_{j+1}/2) < 0\,, \\ (1-q) & \text{otherwise}\,, \end{cases} \tag{18}$$

where

$$q = \frac{\Gamma_q\,\delta\tau}{\omega_0} \qquad p_{0j} = \exp\left(-\frac{\widetilde{v}_0}{\frac{1}{2}(v_j + v_{j+1})}\right), \quad \text{and} \qquad p_{1j} = \exp\left(-\frac{\widetilde{v}_1}{\frac{1}{2}(v_j + v_{j+1})}\right). \tag{19}$$

Here Γ_q is the rate of quasiparticle poisoning, and $\widetilde{v}_0 = V_0/R_{\text{eff}}I_m$ and $\widetilde{v}_1 = V_1/R_{\text{eff}}I_m$ are the dimensionless voltage scales for LZ tunneling between branches and to the continuum, respectively.

We estimate the PSD of $v_c(\tau)$ by applying Welch's method [18] to the simulated time series. The result is then averaged over several independent simulations with the same parameter values. The simulation step size $\delta\tau$ is chosen by requiring that the computed PSD not change significantly when $\delta\tau$ is made smaller (see Supplementary Figure 16).

In the case of a non-topological short junction containing a single open channel with transmission eigenvalue $\mathcal{T}$ (and thus hosting a single Andreev bound state), the update rules are still given by Eqs. (17), (18) and (19), but with

$$s(\phi) = \left(\frac{1+\sqrt{1-\mathcal{T}}}{2}\right)\frac{\sin\phi}{\sqrt{1-\mathcal{T}\sin^2(\phi/2)}}\,, \tag{20}$$

where $\widetilde{v}_0 = V_0/R_{\text{eff}}I_0$, and $p_{1j} = 1$. In the limit $\mathcal{T} \ll 1$ with I_0 fixed, one has $s(\phi) \approx \sin\phi$, and $\widetilde{v}_0 \to \infty$ (so that $p_{0j} \approx 0$).

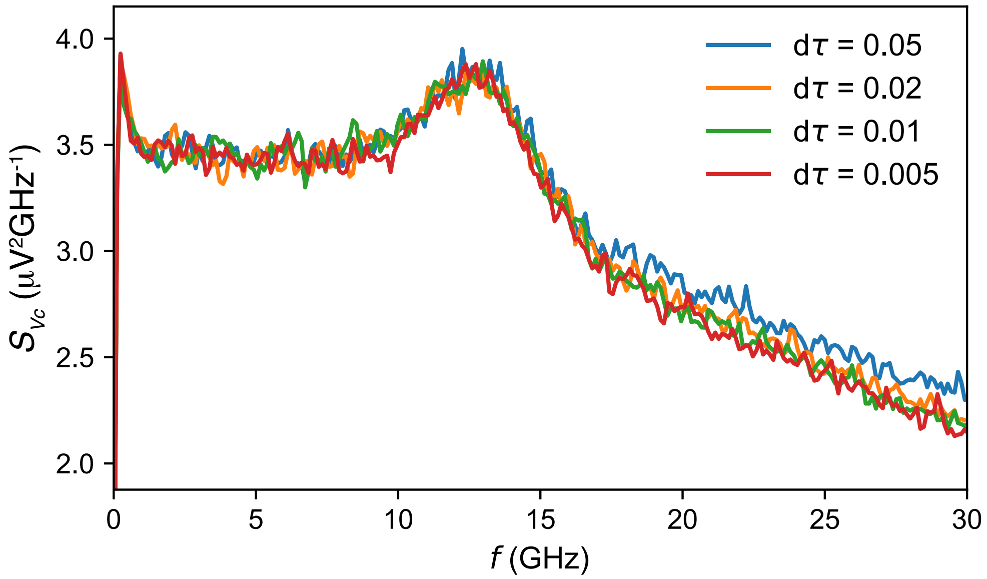

Supplementary Figure 16: **Simulation results with varying step sizes.** Estimated PSD of $V_c(t)$ from simulations using the Euler-Maruyama update Eq. (17) with different step sizes $\delta\tau$, plotted versus frequency f. These results are for a trivial tunnel junction ($I_J(\phi) = I_0 \sin\phi$); circuit parameters are fixed at $r = 2.4\,\mathrm{k\Omega}$, $R_{\mathrm{NW}} = 50\,\mathrm{k\Omega}$, $R = 0.5\,\mathrm{k\Omega}$, $C = 10\,\mathrm{fF}$, $C_c = 400\,\mathrm{fF}$, $I^0_{\mathrm{NW}} = 8\,\mathrm{nA}$, $T = 150\,\mathrm{mK}$ (recall, however, that only the dimensionless ratios $\alpha, \gamma, Q, \Gamma$ appear in the equations of motion) and $\Gamma_q = 100\,\mathrm{MHz}$. The estimated PSD is essentially independent of simulation step size for $\delta\tau \lesssim 0.02$ (the deviations at larger $\delta\tau$ are likely due to aliasing of the spectrum above the Nyquist frequency). Similar results are obtained in the topological case, and for various other circuit parameter values. The simulations that were used to generate Fig. 4 and Supplementary Figure 17 were performed with a step size of $\delta\tau = 0.02$.

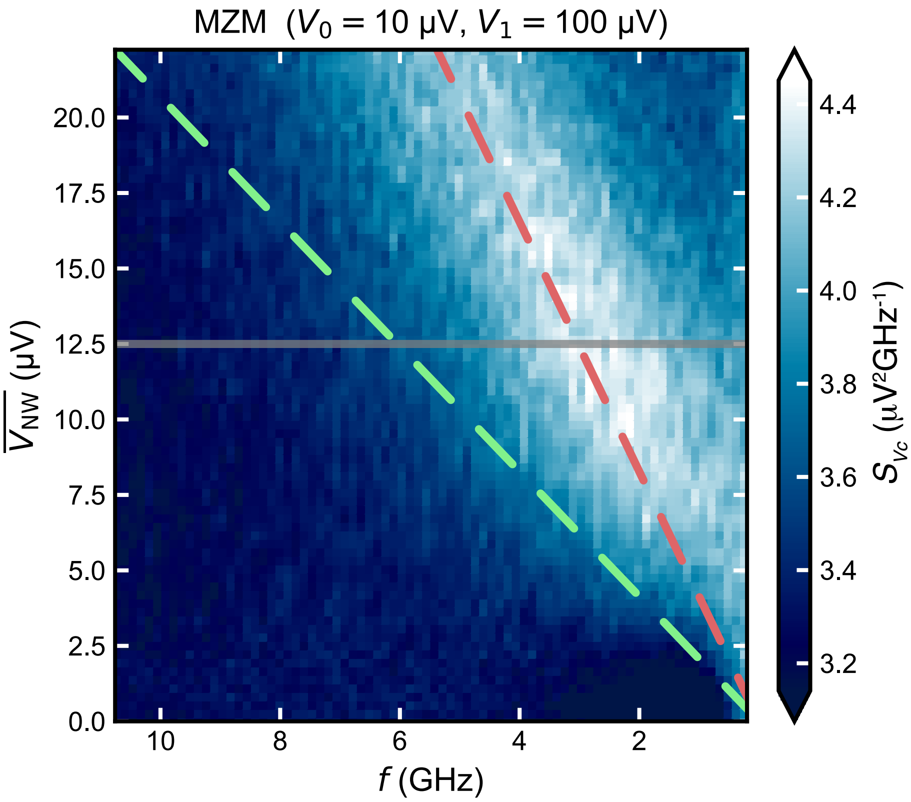

Supplementary Figure 17: **Phase diffusion regime.** Numerically computed PSD of $V_c(t)$ for a topological junction with the indicated voltage scales for LZ tunneling between branches ($V_0 = 10\ \mu$V) and to the continuum ($V_1 = 100\ \mu$V). This plot corresponds to a zoom in of the lower right corner of Fig. 4c (all other circuit parameters are as specified in the Fig. 4c caption). Dashed green and red lines show the frequency of ideal Josephson radiation corresponding to $e^\star = 2e$ and $e^\star = e$, respectively. The horizontal gray line marks the upper boundary of the estimated phase diffusion region.

Derivation of power spectrum for fixed junction voltage

If the Majorana junction is biased by a constant external voltage $V > 0$, then the phase difference across the junction increases linearly with time: $\phi(t) = \phi_0 + 2\pi\eta t$, where $\eta = (2e/h)V$. With the choice $\phi_0 = \pi$, the supercurrent at time t is given by

$$I(t)/I_m = \xi_{\lfloor \eta t \rfloor} \cos(\pi(\eta t - \lfloor \eta t \rfloor))\,, \tag{21}$$

where $\lfloor z \rfloor$ denotes the integer part of z, and the random variable $\xi_n = \pm 1$ specifies the branch during the time interval $\eta t \in [n, n+1)$, i.e. between two LZ tunneling events. The

probability vector

$$X_n = \begin{pmatrix} x_n^+ \\ x_n^- \end{pmatrix} = \begin{pmatrix} P(\xi_n = +1) \\ P(\xi_n = -1) \end{pmatrix} \tag{22}$$

evolves according to

$$X_{n+1} = \begin{pmatrix} 1-p & p \\ p & 1-p \end{pmatrix} X_n \,, \tag{23}$$

where $p = e^{-V_0/V}$ is the LZ tunneling probability.

The supercurrent power spectral density is defined as

$$S_I(\omega; V, V_0) = \lim_{T\to\infty} \frac{1}{T} \int_0^T dt \int_0^T dt' \, \langle I(t) I(t') \rangle \, e^{i\omega(t-t')} \tag{24}$$

(in this section, T is time rather than temperature). Using Eq. (21) and changing integration variables to $\tau = \eta t$,

$$S_I(\omega; V, V_0) = \lim_{T\to\infty} \frac{I_m^2}{\eta^2 T} \int_0^{\eta T} d\tau \int_0^{\eta T} d\tau' \, \left\langle \xi_{\lfloor \tau \rfloor} \xi_{\lfloor \tau' \rfloor} \right\rangle \cos(\pi(\tau - \lfloor \tau \rfloor)) \cos(\pi(\tau' - \lfloor \tau' \rfloor)) \, e^{i(\omega/\eta)(\tau - \tau')} \,. \tag{25}$$

Taking $\eta T = N$ to be a positive integer, we can write

$$\begin{aligned} S_I(\omega; V, V_0) &= \lim_{N\to\infty} \frac{I_m^2}{\eta N} \sum_{n=0}^{N-1} \sum_{n'=0}^{N-1} \int_0^1 d\tau \int_0^1 d\tau' \, \langle \xi_n \xi_{n'} \rangle \cos(\pi\tau) \cos(\pi\tau') \, e^{i(\omega/\eta)(n+\tau-n'-\tau')} \\ &= \lim_{N\to\infty} \frac{I_m^2}{\eta N} \left[\sum_{n=0}^{N-1} \sum_{n'=0}^{N-1} \langle \xi_n \xi_{n'} \rangle \, e^{i(\omega/\eta)(n-n')} \right] \left| \int_0^1 d\tau \cos(\pi\tau) e^{i(\omega/\eta)\tau} \right|^2 . \end{aligned} \tag{26}$$

One has

$$\left| \int_0^1 d\tau \cos(\pi\tau) e^{i2\pi x\tau} \right|^2 = \left| \frac{x(1+e^{i2\pi x})}{2\pi(x^2 - 1/4)} \right|^2 = \frac{x^2(1+\cos 2\pi x)}{2\pi^2(x^2-1/4)^2} \,. \tag{27}$$

In the remaining term, homogeneity of the random process ξ ensures that $\langle \xi_n \xi_{n'} \rangle = \langle \xi_{n-n'} \xi_0 \rangle$. We define

$$f_n = \langle \xi_n \xi_0 \rangle \,. \tag{28}$$

Clearly, $f_0 = 1$. For $n \geq 1$, we have

$$f_n = P(\xi_n = \xi_0) - P(\xi_n = -\xi_0) \,. \tag{29}$$

By conditioning on ξ_{n-1},

$$P\big(\xi_n = \pm\xi_0\big) = (1-p) P\big(\xi_{n-1} = \pm\xi_0\big) + p P\big(\xi_{n-1} = \mp\xi_0\big) \,. \tag{30}$$

Therefore, f satisfies the recursion

$$f_{n+1} = (1-2p) f_n \,. \tag{31}$$

Since $f_0 = 1$, the solution is simply

$$f_n = (1-2p)^n \,. \tag{32}$$

Thus,

$$\begin{aligned}
\lim_{N\to\infty} \frac{1}{N} \sum_{n=0}^{N-1} \sum_{n'=0}^{N-1} \langle \xi_n \xi_{n'} \rangle \, e^{i2\pi x(n-n')} &= \sum_{k=0}^{\infty} \langle \xi_k \xi_0 \rangle \left(e^{i2\pi xk} + e^{-i2\pi xk} \right) - 1 \\
&= \sum_{k=0}^{\infty} (1-2p)^k \left(e^{i2\pi xk} + e^{-i2\pi xk} \right) - 1 \\
&= \frac{1}{1-(1-2p)e^{i2\pi x}} + \frac{1}{1-(1-2p)e^{-i2\pi x}} - 1 \\
&= \frac{1-(1-2p)^2}{1+(1-2p)^2 - 2(1-2p)\cos 2\pi x} \,.
\end{aligned} \tag{33}$$

Using Eqs. (27) and (33) in Eq. (26), we obtain

$$S_I(\omega = x \cdot 2\pi\eta; V, V_0) = \frac{I_m^2}{2\pi\eta} \left[\frac{x^2(1+\cos 2\pi x)}{\pi(x^2 - 1/4)^2} \right] \left[\frac{1-(1-2p)^2}{1+(1-2p)^2 - 2(1-2p)\cos 2\pi x} \right] . \tag{34}$$

Recalling that $p = e^{-V_0/V}$ and $\eta = 2eV/h$, this yields Eqs. (6) and (7).

Derivation of circuit equations

Kirchhoff's laws, applied to the circuit of Supplementary Figure 11, require

$$I - I_J - I_1 + I_n = I_2 \,, \qquad I_2 - I_3 + I_{n2} = I_4 \,, \qquad V = \widetilde{V}_1 + V_c + \widetilde{V}_2 \,. \tag{35}$$

The current-voltage relations for the linear passive elements are

$$I = \frac{V_s - V}{r} \,, \qquad I_1 = \frac{V}{R_{\rm NW}} \,, \qquad I_2 = C_c \frac{d\widetilde{V}_1}{dt} = C_c \frac{d\widetilde{V}_2}{dt} \,, \qquad I_3 = \frac{V_c}{R} \,, \qquad I_4 = C \frac{dV_c}{dt} \,. \tag{36}$$

The ideal Josephson junction element is described by Eqs. (8) and (9):

$$\frac{d\phi}{dt} = \frac{2e}{\hbar} V(t) \,, \qquad I_J(t) = I_0 \sin\phi(t) \,. \tag{37}$$

Using these relations in Eq. (35), we obtain

$$C_c \frac{d\widetilde{V}_1}{dt} = \frac{V_s - V}{r} - I_0 \sin\phi - \frac{V}{R_{\rm NW}} + I_n \,, \qquad C \frac{dV_c}{dt} = C_c \frac{d\widetilde{V}_1}{dt} - \frac{V_c}{R} + I_{n2} \,, \qquad \frac{dV}{dt} = \frac{dV_c}{dt} + 2\frac{d\widetilde{V}_1}{dt} \,. \tag{38}$$

Therefore,

$$C \frac{dV}{dt} = (C_c + 2C)\frac{d\widetilde{V}_1}{dt} - \frac{V_c}{R} + I_{n2} = \left(1 + \frac{2C}{C_c}\right)\left[\frac{V_s}{r} - \left(\frac{1}{r} + \frac{1}{R_{\rm NW}}\right) V - I_0 \sin\phi + I_n\right] - \frac{V_c}{R} + I_{n2} \tag{39}$$

and

$$C \frac{dV_c}{dt} = \frac{V_s}{r} - \left(\frac{1}{r} + \frac{1}{R_{\rm NW}}\right) V - I_0 \sin\phi + I_n - \frac{V_c}{R} + I_{n2} \,. \tag{40}$$

Eqs. (39) and (40), together with Eq. (8), constitute the relevant equations of motion. Expressing them in terms of the quantities defined in Eq. (11), we arrive at the dimensionless equations of motion Eq. (12).

SUPPLEMENTARY REFERENCES

[1] Mourik, V. *et al.* Signatures of Majorana fermions in hybrid superconductor-semiconductor nanowire devices. *Science* **336**, 1003–1007 (2012).

[2] Das, A. *et al.* Zero-bias peaks and splitting in an Al-InAs nanowire topological superconductor as a signature of Majorana fermions. *Nature Physics* **8**, 887–895 (2012).

[3] Albrecht, S. *et al.* Exponential protection of zero modes in Majorana islands. *Nature* **531**, 206–209 (2016).

[4] Zhang, H. *et al.* Quantized Majorana conductance. *Nature* **556**, 74 (2018).

[5] Pientka, F., Kells, G., Romito, A., Brouwer, P. W. & von Oppen, F. Enhanced zero-bias Majorana peak in the differential tunneling conductance of disordered multisubband quantum-wire/superconductor junctions. *Phys. Rev. Lett.* **109**, 227006 (2012).

[6] Prada, E., San-Jose, P. & Aguado, R. Transport spectroscopy of NS nanowire junctions with Majorana fermions. *Phys. Rev. B* **86**, 180503 (2012).

[7] Stanescu, T. D., Tewari, S., Sau, J. D. & Das Sarma, S. To close or not to close: The fate of the superconducting gap across the topological quantum phase transition in Majorana-carrying semiconductor nanowires. *Phys. Rev. Lett.* **109**, 266402 (2012).

[8] Rainis, D., Trifunovic, L., Klinovaja, J. & Loss, D. Towards a realistic transport modeling in a superconducting nanowire with Majorana fermions. *Phys. Rev. B* **87**, 024515 (2013).

[9] Deblock, R., Onac, E., Gurevich, L. & Kouwenhoven, L. P. Detection of quantum noise from an electrically driven two-level system. *Science* **301**, 203–206 (2003).

[10] Efron, B. & Tibshirani, R. Bootstrap methods for standard errors, confidence intervals, and other measures of statistical accuracy. *Statistical Science* **1**, 54–75 (1986).

[11] Pikulin, D. I. & Nazarov, Y. V. Phenomenology and dynamics of a Majorana Josephson junction. *Phys. Rev. B* **86**, 140504 (2012).

[12] Landau, L. D. Zur Theorie der Energieubertragung. II. *Phys. Z. Sowjetunion* **2**, 1–13 (1932).

[13] Zener, C. Non-adiabatic crossing of energy levels. In *Proceedings of the Royal Society of London A: Mathematical, Physical and Engineering Sciences*, vol. 137, 696–702 (The Royal Society, 1932).

[14] Demkov, Y. N. & Osherov, V. Stationary and nonstationary problems in quantum mechanics that can be solved by means of contour integration. *Sov. Phys. JETP* **26**, 1 (1968).

[15] Houzet, M., Meyer, J. S., Badiane, D. M. & Glazman, L. I. Dynamics of Majorana states in a topological Josephson junction. *Phys. Rev. Lett.* **111**, 046401 (2013).

[16] Kautz, R. L. & Martinis, J. M. Noise-affected I-V curves in small hysteretic Josephson junctions. *Phys. Rev. B* **42**, 9903 (1990).

[17] Gardiner, C. W. *Handbook of stochastic methods for physics, chemistry and the natural sciences*. Springer Series in Synergetics (Springer, 2004), 3 edn.

[18] Welch, P. The use of fast Fourier transform for the estimation of power spectra: a method based on time averaging over short, modified periodograms. *IEEE Transactions on audio and electroacoustics* **15**, 70–73 (1967).
